# Reflectivity relates differently to pro sociality in naïve and strategic subjects

**DOI:** 10.1038/s41598-021-91960-3

**Published:** 2021-06-17

**Authors:** Francesca Pancotto, Simone Righi

**Affiliations:** 1 University of Modena and Reggio Emilia, Viale Allegri 9, 42121 Reggio Emilia, Italy; 2 University Ca’Foscari of Venice, Fondamenta S. Giobbe, 873, 30121 Venezia, Italy

**Keywords:** Cultural evolution, Evolution, Applied mathematics

## Abstract

Is pro sociality a natural impulse or the result of a self-controlled behavior? We investigate this issue in a lab in the field experiment with participants from the general adult population in Italy. We find two key results: first, that there is a positive relationship between pro sociality and strategic reasoning. Second, that reflectivity relates to lower pro sociality but only among strategic subjects, indicating that the intuitive view of pro sociality is valid only among strategic individuals. Non-strategic individuals are instead intuitively selfish. We surmise that these results emerge due to a common cognitive root between strategizing and pro sociality, namely empathy.

Procedures Pro sociality is defined as a voluntary behavior intended to benefit another person, group, or the society as a whole. It is one of the most remarkable qualities of human behavior yet still puzzling for scholars. Motivations proposed for pro social behavior are many: empathy, concern for the welfare of others, care for reputation or personal social status, the expectation of a social reward or pure altruism^1^. The nature of the cognitive mechanism activating pro social behavior is still subject to debate. The dual system of cognitive processing suggests that human choices are governed by two systems: a slow, effortful and calculated system and an intuitive, fast, and automatic one. Reflectivity (or cognitive reflection) has been defined^2^ as the ability or disposition to resist reporting the response that first comes to mind. This disposition is frequently measured trough a set of questions whose correct answers are not those who first come to mind. Subjects who respond correctly to these questions are identified as using the slow, calculated (reflective) system of reasoning; those who respond incorrectly are instead identified as using the fast, intuitive system of reasoning. However, it is not yet clear whether pro social choices are generated by the calculated or the intuitive system^3^, and if the key motivations differ for different individuals.

The traditional intuitive-selfishness hypothesis (ISH) postulates that humans are basically selfish and that they need to exert reflective control to act pro socially^4^. Pro sociality would then be the outcome obtained through cognitive efforts. More recently, a number of contributions have put forward the Social Heuristics Hypothesis (SHH) which questioned this view, suggesting instead that humans are naturally pro social and that, in order to behave selfishly, they need to exert reflectivity^5–7^. The SHH asserts that people apply intuitive or reflective prosocial heuristics following the history of cooperation experience. Pro social behaviors become convenient in real life when subjects can reasonably anticipate collaborative rather than selfish behaviors thanks to repeated interactions and the possibility to exert sanctions. SHH suggests that pro social attitude is then internalized becoming an heuristic response applied extensively by people engaging in social dilemmas thus also explaining pro sociality in one-shot anonymous decision contexts where cognitive reflection would rather predict a selfish choice^6^. SHH further implies that intuition should favor pro sociality rather than selfishness in cultures where social experience and institutional framework allow people to develop the habit of pro sociality.

There is a large and growing number of experimental contributions that test these two opposite hypotheses using various proxies to measure intuitive versus reflective behavior and/or trying to exert such behavior through various experimental manipulations. Carefully reviewing the experimental approaches in the literature, it is possible to identify both similarities and differences in design that might justify the different outcomes and that help guiding further research. Indeed, while finding opposite results, both SSH and the ISH, traditionally relied heavily on two manipulations to assess whether pro sociality is intuitive or consequence of reflection: time pressure (versus delay) and the cognitive load (or ego depletion).

Using time pressure to force subjects to rely on intuitive behavior, some authors find that it increases pro sociality^6,8–14^. Other contributions find instead that—in line with the ISH—when the time pressure becomes very intense^15–17^, it decreases cooperation. Exerting heavy time pressure^15^, find that cooperation is a cognitively intensive process^17^, that egoistic decisions are faster in a modified dictator game, and^16^ that if subjects are put in the conditions to avoid knowing that their behavior is self-serving this increases anti social behavior. The role of time is also studied by controlling pro social attitudes using response time^18–20^, with results in support of the SSH. In particular, Lohse et al^20^ find that people which answer more slowly tend to be more pro social when asked to donate to a pro environmental cause. Overall, the intensity of the time pressure seems to play a crucial role in determining the direction of results.

Diverging results are found also for cognitive load manipulation, with some experiments finding support for the SHH prediction of an increase in pro sociality under heavy load^21–24^, and others supporting ISH prediction of a negative effect of cognitive load on pro sociality^15,25^. On this second group, given that cognitive load leads to depletion, Achtziger et al^25^ manipulates self-control resources and find that depleted dictators give considerably less than non-depleted ones. A possible reason for these different findings might be found in small differences in experimental designs. The cognitive tasks used by^15,25^(i.e. Stroop task, the e-hunting task, and give-the-wrong-answer task) differ from those largely adopted in the SHH literature (i.e. remembering letters and numbers and doing simple operations with them). This difference might influence the overall levels of cognitive depletion, and—if some non-monotonicity exists—the sign of the correlation with pro sociality. Indeed, the tasks used in^15,25^ are more complex than those largely adopted by the SHH literature, as noted by^26^, which also find significantly depletion for the former.

A significant amount of contributions, while using the aforementioned manipulations, do not find any relationship between intuition and pro sociality^27–31^. Beside these traditional manipulations, other approaches have been attempted including asking participants to use their intuition (induction manipulation), to rely on their emotions (emotion induction), or to recall times in which decisions were done more or less intuitively (recall induction) in their decisions. This extensive literature has been summarized in several reviews and meta-analyses. While overall they find evidence of a positive effect of intuition on cooperation^32–34^, the observed effects have been shown to depend on the presence of studies including the emotion induction manipulations, without which the effect of intuition on cooperation disappears^35^. Finally, using less standard approaches, cooperation is found to increase with attention to other’s payoff (identified through the eye movements in the context of an eye-tracking experiment^36^) and with personality traits linked to self-control^37^.

The diverseness of results emerging from small changes in the experimental setup and the potential interactions with individual heterogeneity, lead us to propose an experimental test using games to characterize individuals according to their types (as elicited by the games) rather than by their reaction to experimental manipulations. Indeed, the reason for the multiplicity of results might be that the relationship between intuition and pro sociality has a different sign for different people. In other terms it is possible to hypothesize that both intuitively selfish and intuitively pro social individuals exist. If both intuitively selfish and intuitively prosocial people exists the question becomes then, what could differentiate the two groups. As noted by Brañas-Garza et al^38^, the ability to overcome instinctive reactions in favour of more ponderate ones is a precondition for strategizing, but the latter implies also some other element of thought. Indeed, strategizing implies also internalizing expectations about others’ behaviour^39^. If this is the case, then strategizing should be taken in consideration as a mediator between reflectivity and pro sociality. To be clear, while both strategizing behavior and reflectivity characterize the cognitive ability of individuals, they point to different aspects of it. On the one side, the ability to strategize determines the measure in which a player is able to incorporate other players’ behaviour in their own decision-making processes^39^. On the other side, reflectivity points to the propensity of an individual to reject automatic response to situations in favour of more elaborate and deliberative thought. Thus, the latter prescinds from strategic situations, although it constitutes one of the requirements for rational decision making in strategic situations.

In this paper, we thus run a lab-in-the field experiment where we measure pro sociality with a distribution game (DG)^40^ and impulsive versus reflective behaviors using an extended version of the popular Cognitive Reflection Test^2^. We further control for the strategic ability of subjects using the guessing game to assess ‘whether and how a player’s mental process incorporates the behavior of the other players in conscious reasoning.’^41^. As a further improvement on the current methods we elicit both guessed values and declared motivation of the choice. The choice of the DG over strategic games alternatives (e.g. the trust game, public good game, ultimatum game) is dictated by the need to keep strategic considerations outside of the individual decisions about pro sociality so that the latter can then be related more neatly with strategic reasoning elicited through the guessing game.

To our knowledge this is the first paper that tests the interaction of these three behavioral motivations in an experimental setting. There is however a literature assessing the dyadic relationship between pairs of these three components with mixed results. Some studies analyze the connection between strategic behavior and pro sociality, without controlling for intuitive versus reflective behavior, and find a positive relation between strategic behavior and selfishness^42,43^ or no significant relationship^44^. In turn, without controlling for strategic reasoning, there are several studies that—in line with our approach—use CRT as a tool to classify participants and to assess the relationship between their ability to engage in cognitive reflection and pro sociality. The results of this type of studies are inconclusive, indeed while some find a positive relation between the CRT and pro sociality (trust and social efficiency)^13,45,46^, some that find a negative relationship^47–50^.

With our experimental analysis, we find that a crucial role in the understanding of what explains pro sociality is played by the interaction between strategic reasoning and reflectivity. Strategic subjects are intuitively pro social, i.e. it takes an explicit act of self-control, measured in our experiment by the level of reflectivity, to exert a selfish behavior from strategic subjects. The opposite holds for non-strategic subjects, which are impulsively selfish. Our interpretation is that the impulsive pro sociality of strategic subjects may be linked to an ability to perceive intuitively the relationship between their action and the effect on others, which we surmise as sharing a common cognitive root with strategic reasoning. We speculate that this common root is empathy, as suggested by^51,52^. Being a natural response to individual perception of others’ feelings, empathy possesses a natural commonality with strategic reasoning^53^ and can help us predict pro sociality^54^. Our results are robust controlling for age - negatively related to pro sociality - and students status - which is positively related to pro sociality (as in^55^). Our contribution qualifies the current debate in the understanding of the cognitive roots of pro social behavior, providing an important framing in which the impulsive pro social response could be introduced, i.e. strategic reasoning.

Finally, it is important to mention that the subjects in our lab-in-the-field experiment are not standard undergraduate students participants but are drawn from the general population recruited in three different locations in Northern Italy (see *Methods* section). Our decision to employ lab-in-the-field experiment stems from the observation that, while SHH has been studied through both online (on Amazon Mechanical Turk, AMT) and in-presence experiments, ISH has been studied (with the notable exception of^15^) mainly with in-presence experiments. Furthermore, most ISH analyses involve student-only setups, while SSH involved both general population (on AMT) and students-only samples. This could in principle condition ISH results, as students are more likely, with respect to the general population, to be instructed and conditioned to think in selfish terms due to their exposure to at least basic form of rational economic thinking^56^. Finally, a more comprehensive sample, with enhanced internal heterogeneity, best fits our analysis aimed at classifying and correlate individuals according to their types.

## Experimental design

Our experimental setup comprises of a series of tasks presented in consecutive order to the same subjects, without giving a feedback on the outcome of the single parts before the end of the whole experiment. The tasks performed are summarized in Table 1, while the full instructions are available in Sect. S7 of Supplementary Information (SI).

**Table 1:** Sequence of tasks in each session.

INCENTIVIZED TASKS	Public Goods Game (PGG)
	Guessing Game (GG)
	Distribution Game (DG)
NON-INCENTIVIZED TASKS	Comments about choice in GG (C-GG)
	Survey (S)
	Cognitive Reflection Test (CRT)
TIME LINE:	PGG $->$ GG $->$ C-GG $->$ DG $->$ S $->$ CRT

PGG sessions included two variants of the Public Good Game: Standard and Strategy Method. The Results concerning these games are discussed in^57^.

The results of the PGG are discussed in^57^ and in supplementary material as robustness check. The first task analyzed in this paper, the guessing game (GG^41^), is aimed at measuring the depth of strategic reasoning. Data are collected on two levels, the actual choice and the declared motivation of the choice. For the actual choice, subjects must choose a number between 0 and 100, knowing that the winner of the game is the subject picking the closest number to $p=2/3$ of the average of the group components’ choices. Then, after finalizing their own choice, subjects are asked to write down (on paper) the motivation for the choice; i.e., a strategy, a rule of thumb or a reasoning procedure - if any was used - thanks to which they made their choice. The first part of the assessment - the actual choice game - is economically incentivized while the second is not.

The second task analyzed in this paper is a distribution game (DG) or four-players dictator game, taken from^40^, in which each participant has to select a preferred allocation, among three different options, of the same amount of experimental points to anonymous components of his/her group. The participants are informed that the computer will select only one of each group to be the dictator and that this selection will determine the payoffs of everyone according to the preferences expressed by the selected subject. All the other choices will be payoff irrelevant after this extraction. This task is economically incentivized. Parameters values are chosen in order to stress the opposition between a selfish and a prosocial choice. The pro social choice (Choice C in Table 2) is selected according to Fehr and Schmidt (1999) theory of equity (F&S in the Table)^58^, from Bolton and Ockenfels(2000) (known as ERC: A Theory of Equity, Reciprocity, and Competition)^59^ and minimax criteria^40^. Choice C is pro social because it maximizes both ERC and F&S criteria (see the notes of Table 2 for their formalization), the payoff of the poorest (minimax) and the average income of the group. Choice A is selfish because it presents the highest income for the dictator and the highest variance in group payoffs. Choice B lays in the middle between the other two. The actual choice screens presented to subjects are shown in Fig. S11 in SI. We propose alternative allocations of the same income among four participants (total income is always equal to 96) in order to eliminate the efficiency motivation. Indeed the latter is beside the scope of the present study and—being a salient motivation—could distort results related to pro sociality^40^. The individual pro sociality has been measured by various games in the literature such as the PGG^6,9–13,15,18,20,27,28,36,37^, the Ultimatum Game^8,13,14^ and the DG^7,11,13,14,16,17,21,22,25,31^. The choice of adopting the DG as the game to measure the pro sociality of individuals, stems from the observation of^13,60^ that other social games such as the PGG or the Ultimatum game, involve a degree of strategic interactions among subjects, and decisions in those games involves expectations about others’ behaviour. The DG isolates the choice of the individual from other considerations, and it is particularly advisable given that we are measuring the subjects’ strategic abilities with the GG.

**Table 2:** Parameters of the distribution game (DG).

Player	Choice A	Choice B	Choice C
Person 1	51	45	42
DICTATOR	30	27	24
Person 3	9	15	18
Person 4	6	9	12
Total Income	96	96	96
Criteria			
Group Variance	328.5	189	126
Bolton-Ockenfels (ERC)	− 6.25	− 3.13	0
F&S Strict	− 22	− 16	− 12
Minimax	6	9	12
Average income of other group members	22	23	24

Group Variance is the variance of the payoffs of each choice. Bolton & Ockenfels(ERC) is calculated as $ERC = - abs\left( {\frac{{Dictator\;Payoff}}{{96}} - \frac{1}{4}} \right)$. $F \& S \,Strict = -\frac{1}{4} \sum abs (Payoff_i-Dictator\,Payoff)$. Minimax is the value of the minimum payoff among the four components of the group in each presented possible choice. Average is the simple average of the payoffs of the group in each choice excluding the dictator.

Finally, cognitive reflection is measured with an extended and improved version of^2^’s Cognitive Reflection Test (CRT) based on^61^. This extended version overcomes important limitations of the original. First of all, new items are easier to understand, overcoming the intrinsic limit of the original test concerning the reliability of the results for participants with lower education levels. Second, the eight items extended scale (rather than 3) permits the construction of a wider scale of responses upon which to classify the observed population. Finally, the original test has now become very popular and the solutions are easily available in the web: the new items contribute to eliminate false positive answers due to informed participants who already know the results. The english version of the test is reported in Sect. S9 of SI.

Tasks were run with groups of four subjects, that were randomly and anonymously matched by the computer before each task. Before each task, each participant had also to respond to two training questions in order to increase tasks understanding. Taking into account also the fact that the population of participants was quite diverse (See Tables 9 and S29 for details), clarity was further enhanced by rendering choice screens with visual vignettes and examples (See Fig. S10 and S11). After the completion of each task, subjects were informed that they would be reassigned to a new random and anonymous group of four participants.

The experimental procedures and the structure of the sample are detailed in the Methods section.

## Characterization of Strategies

The objective of this article is to evaluate pro sociality in connection with strategic reasoning and reflectivity. Provided that the structure of the experimental design comprises three tasks presented to the same pool of subjects, we classify the basic behavioral patterns of individuals according to their pro sociality, level of strategic reasoning and reflectivity.

### Distribution game

The choices in the Distribution Game (DG), can be classified according to the level of pro sociality. Payoff maximizing game-theoretic agents should always pick the option that maximizes personal profit, i.e. choice A. However, the actual choices that our participants made during the experiment, correspond to the frequencies in Table 3. Our subjects are in large part pro social, consistently with the literature^62^.

**Table 3:** Distribution game, number and proportion of people making each choice.

Interpretation	Choice	#	%
Self-interested	Choice A	39	22%
Middle	Choice B	34	19%
Pro social	Choice C	103	59%

### Guessing Game (GG and C-GG)

Here we analyze data of the guessing game, i.e. the actual choice (GG) and the comments about the choice (C-GG) and we discuss the categorization of individual between strategic and naïve. In this game, for a parameter value of $p\in [0,1)$, there is a unique Nash equilibrium in which all players choose 0. The value 0 chosen by all players is also the strategy combination that survives the procedure of infinitely repeated simultaneous elimination of weakly dominated strategies. This is because a rational player is supposed to eliminate weakly dominated strategies, which are values that multiply $p=2/3$ by 100 or larger (i.e, in the interval (100*p*, 100]). Following this procedure, if the rational player believes the other players to be rational as well, he should expect nobody to choose values in the interval (100*p*, 100], which would lead to exclude also values in the interval $(100p^{2}, 100]$. The limit of this reasoning process leads to the equilibrium value of 0.

The experimental literature^63^ has found that the reasoning process that appears to describe better actual observed behavior is the Iterated Best Reply model with degenerate beliefs (where degenerate refers to the belief that the choices of all others are at one precise value), known as IBRd. The latter classifies choices according to the depth, or number of levels, of reasoning that each subject is supposed to implement when making his/her decision. Specifically, IBRd postulates that a zero level player chooses randomly in the given interval [0, 100], with the mean being 50. At other levels, it is assumed that every player believes that he/she is exactly one level of reasoning deeper than the rest of players. Therefore, a level-1 player gives best reply to the belief that everybody else is a level-0 player and thus chooses 50*p*, where $p=2/3$. A level-2 player chooses $50p^2$, a level-k player chooses $50p^k$ and so on, up to infinite steps of reasoning where IBRd converges to the rational expectations equilibrium, and yields guessed value zero.

Although the IBRd model postulates that higher steps of reasoning would correspond to lower and lower guesses, limiting at 0 value, we take a simplified version of this model and categorize our responses according to only two levels : level-0 players, that we define *Naïve* and the remaining subjects that we call *Strategic*. *Naïve* subjects are those who make a guess which is equal or higher than 50 while *Strategic* are the remaining subjects. Thus, we codify as strategic only those subjects performing at least one step of reasoning. We choose this classification because the interpretation of low values of the guessing game is not univocal in the literature: a very low guess is not necessarily a rational guess when you expect that all the other participants are not as rational or strategic as you are (i.e. in absence of common knowledge of rationality). Consequently, even a highly sophisticated subject could pick a number higher than 0 following his/her belief that the other players are naïve. Following this idea, we avoid the imposition of a rigid and predetermined structure—the steps of reasoning—to the data while maintaining the idea that a strategic subject must be capable at least to understand that a guess bigger than 50 is irrational or naïve because it is necessarily dominated by any other choice that multiplies 2/3 by the expected average of group choice, which according to the IBRd model is 50.

A further control on the outcome of the guessing game is implemented using the information provided by the written comments about the choice made (C-GG) that we requested to all participants after the completion of the task and without economic incentive. The choice to ask for an explanation to the subjects has been implemented in other designs involving games of strategic reasoning (e.g. Cerigioni et al^64^). The papers that reported the comments of all participants were tracked with their choice in the actual game. The motivation provided for the choice in the guessing game was codified independently by three autonomous persons. The classification, aimed at identifying the real motivation behind the choice in the guessing game, allowed us to codify a variable (*GG-random*) pointing out the subjects that explicitly indicated their guess as random. See Sect. S3 of SI for details about the procedure.

According to our classification, our sample comprises 132 strategic and 44 naïve subjects (Table 4). According to what stated by the participants, out of the 176, 66 choices were made by subjects who explicitly hazarded their guess: interestingly 51 of these are from subjects who performed a strategic choice ($Guess <50$), which suggests that using only the choice of a low value in the guessing game could lead to spurious evaluation of a choice as strategic. Furthermore, 17 people did not write a comment. Coherently, in the analysis that follows, we eliminate these observations from the database. It should be noted that, while most of our results are not dependent upon it (see Sect. S1 of the SI for analyses run on the full dataset), the choice of eliminating random guesses has the byproduct of equalizing the levels of cognitive depletion for what concerns the games played after the GG (whose results could be influenced by different levels of cognitive depletion). Indeed, in our setup being non-strategic means to have made a reasoning process that has led to a dominated or naive choice, and it does not mean avoiding thinking at all. Having eliminated from our sample subjects who responded by chance, we are left with strategic and non-strategic people, both likely to be cognitively depleted at the same level.

An alternative strategy to the one of using the self-reports to identify random guesses, would have requested to change the multiplication parameters as in^41,65,66^ and then verify whether the guesses are proportional to the latter. However, using a sample from the general population requires keeping the experiment shorter, thus reducing the number of task proposed to the subject leading to our choice of using written reports.

**Table 4:** Guessing game: actual choice and self-reported motivation.

N=176		Naïve	Strategic
		($Guess >=50$)	($Guess <50$)
*Actual Choice*	GG	44 (25%)	132 (75%)
*Self-reported motivation*	GG-Random	15 (8.5%)	51 (29%)

*Actual Choice* reports the choices of all participants in the guessing game. *Self-reported motivation* describes data related to the motivation of the choice in the guessing game, where GG-Random describes those subjects that explicitly reported to have hazarded a guess, divided among those making a naïve choice in the GG and those making a Strategic choice in the GG. Percentages are out of N=176.

**Figure 1:**
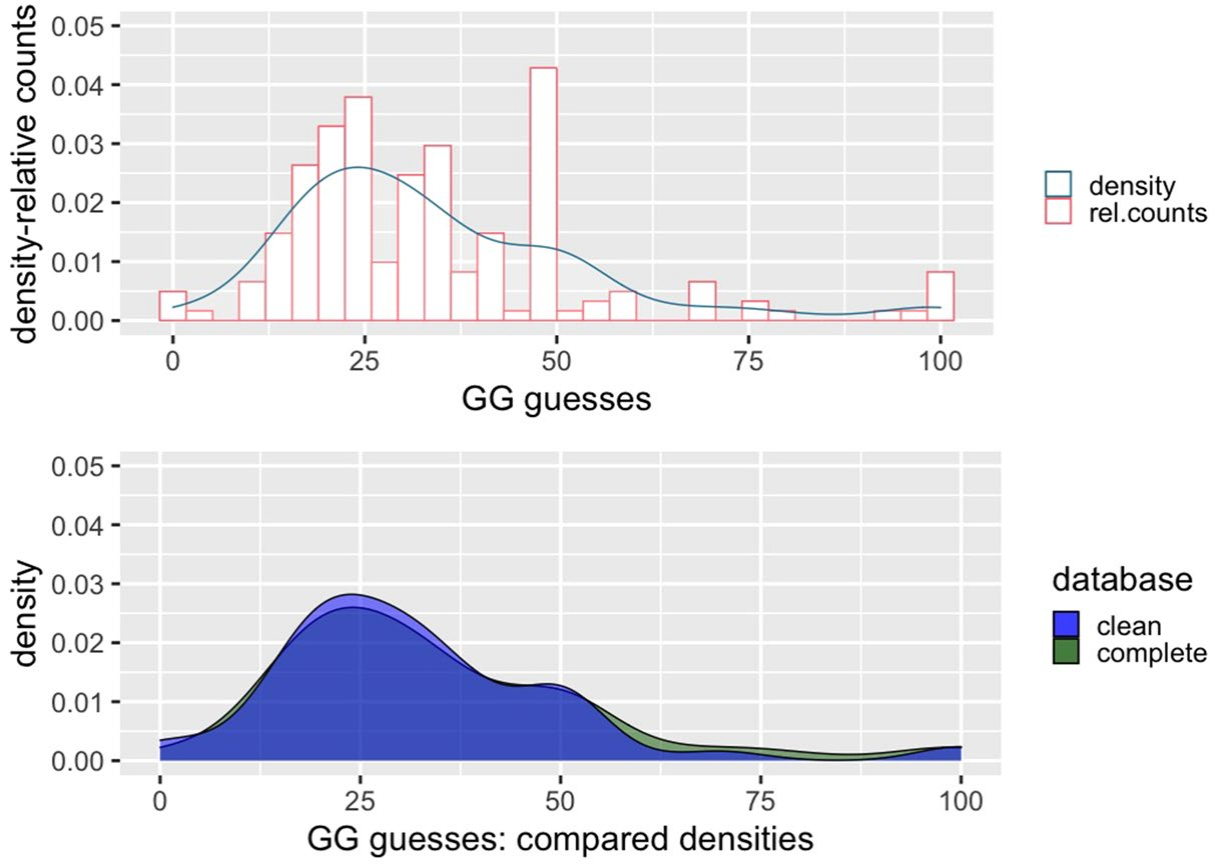
Top: histogram of choices in the guessing game, counts and density. Bottom: Density distribution of choices comparing complete database and clean (i.e., excluding random guesses).

The distribution of choices in the guessing game, which reflects standard results in the literature^41^, is reported in Fig. 1, including also a comparison between complete and clean dataset. Most of the answers are between the values 22 and 33, with a focal point at the value 50.

### CRT

**Figure 2:**
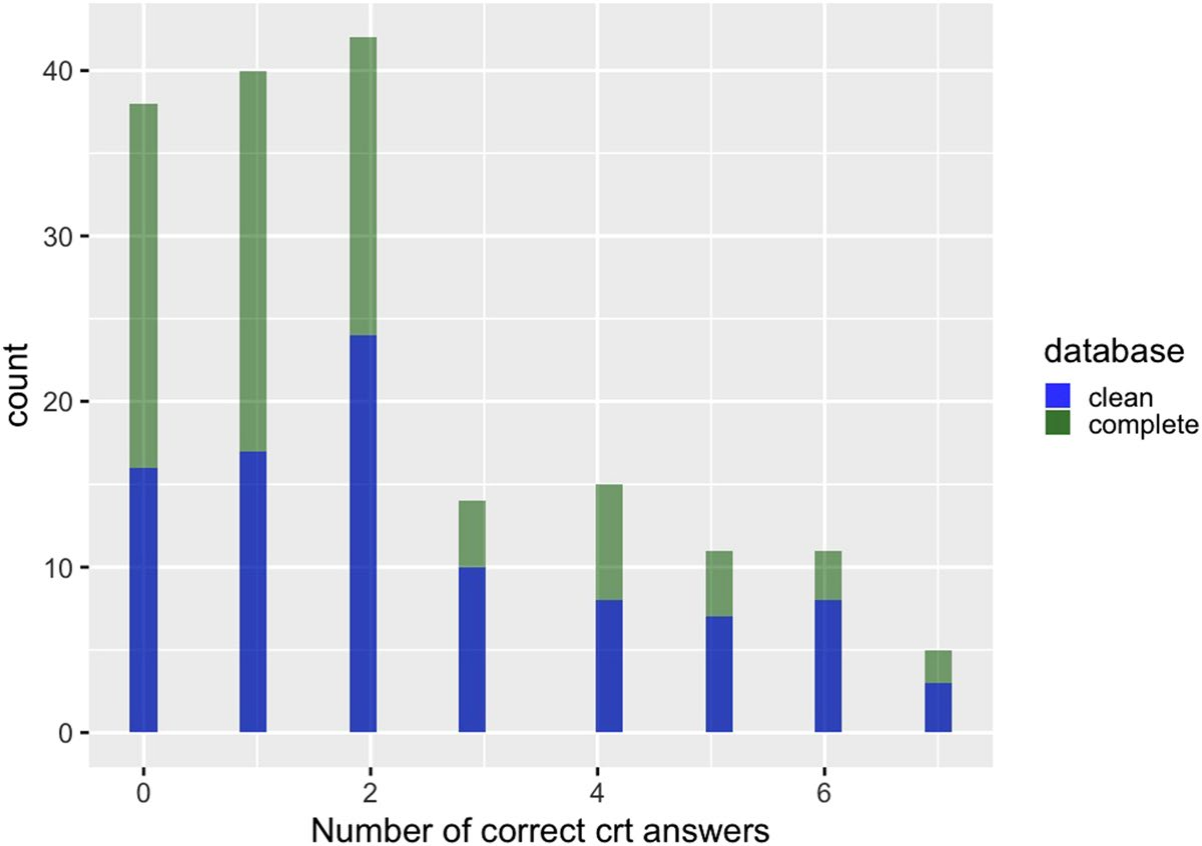
Histogram of choices in the CRT. Comparison between complete and clean database.

In Fig. 2 , we show a histogram with the number of correct answers in the CRT. Most individuals answer correctly to between 0 and 2 questions; while only a few of them answer correctly to three or more. Here, we classify players as *Impulsive* or *Reflective* following two criteria based on the absolute number of correct answers that each participant scored. For the first variable, coded *CRT.DUMMY*, we define as *Impulsive* a subject with zero correct answers, *Middle* a subject with 1 or 2 correct answers, and *Reflective* a subject with three or more correct answers. This classification emerges after observing the frequency of correct answers in the general CRT.

This variable also takes into account the fact that the CRT is a very well known test and consequently some subjects could know by memory the answer to at least one of the three standard CRT questions without even thinking about it. Also, guessing correctly one answer out of eight by chance is a possibility. However, for robustness check, we use also another categorization of the CRT answers, coded as *CRT01*, which defines as *Reflective* a subject that responded correctly to at least one question and as *Impulsive* those who did not respond correctly to any. This second classification is the one most frequently adopted by the literature studying cognitive reflection, although implemented in the three questions version of the test. According to these classifications, we observed the frequencies reported in Table 5.

**Table 5:** Classifications of subjects according to the level of reflectivity in the Cognitive Reflection Test.

CRT.dummy	complete	clean
Impulsive	38	16
(0 correct answers)	(21%)	(17%)
Middle	82	41
(1 or 2 correct answers)	(47%)	(44%)
Reflective	56	36
($>2$ correct answers)	(32%)	(38%)

CRT01	complete	clean
Impulsive	38	16
(0 correct answers)	(21%)	(17%)
Reflective	138	77
($>0$ correct answers)	(79%)	(82%)

## Results

The classification of stylized behavioral patterns performed above allows us to measure the combined effect of strategic reasoning and reflectivity on the pro social choice through an individual-level regression (subscript *i* indicates individuals). Given our research question we estimate the following equation:

1 $$PROSOC_{i} = \alpha + b_{1} {\mkern 1mu} STRAT_{i} + b_{2} {\mkern 1mu} REFL_{i} + b_{3} {\mkern 1mu} STRAT_{i} :REFL_{i} + b_{4} \;CONTROLS_{i} .$$

In Eq. (1) prosoc is a dummy variable that takes values from 1 to 3 corresponding to the choices in the Distribution Game: 1 is the ‘selfish choice’ A , 2 is the choice B, and 3 corresponds to the ‘prosocial’ choice C. strat is a dummy variable with value 1 for subjects providing a strategic answer in the GG (i.e., a guess smaller than 50) and refl is a dummy variable indicating reflective subjects. Reflectivity has been measured with the two variables, CRT01 and CRT.DUMMY. The CRT.DUMMY takes value 1 for subjects with zero correct answers (*Intuitive*), 2 for subjects with 1 or 2 correct answers (*Middle*) and 3 otherwise (*Reflective*). The CRT.01 dummy takes value 1 for Reflective subjects and zero otherwise. The results for the two specifications are qualitatively the same, thus we focus here on the CRT.DUMMY specification (in-depth results for the CRT01 specification are reported in SI, Tables S11 and S12 and in Fig. S9).

The estimation was run including an interaction term that captures the non-linear relationship existing between choices in the GG and the DG and excluding the observations where subjects reported to have guessed randomly in the GG. A number of controls were included, namely: age, occupation status and gender (gathered from the final questionnaire), and the number of training questions that each subject responded correctly before the guessing game task (codified in the variable training question ).

Table 6 reports the results of OLS estimation which indicates positive significant coefficients of both the terms strat and refl, while the interaction term strat : refl is always significant and negative. The power analysis reported at the bottom of the table confirms that the model is able to detect effects which are medium in size. The robustness of results presented is reinforced by their stability when clustering standard errors at the session level (Table S11 and S15) and using Ordered Logit estimation (Tables S16 and S17–S22 for odds ratios). Further robustness is provided by re-classifying individuals as selfish for respondents choosing A or B in the distribution game and prosocial for respondents choosing C (Table S23 and S24 for OLS and Table S25 for Logit estimation). Finally, the regression run without dropping random guesses (Tables S1, S2 and S5) confirms the significance of both the interaction term and of the strat variable, albeit at a lower level due to the added noise caused by individuals which reported having played randomly in the GG. All these additional results are reported and further discussed in SI.

**Table 6:** Pro sociality, strategic reasoning and reflectivity. OLS.

*Dep. Var. : pro sociality*	Model 1	Model 2	Model 3	Model 4
(Intercept)	$$1.10^{*}$$	$$1.43^{***}$$	$$1.94^{**}$$	$$2.34^{***}$$
	(0.65)	(0.48)	(0.75)	(0.61)
STRAT	$$1.67^{*}$$	$$1.29^{**}$$	$$1.64^{*}$$	$$1.26^{**}$$
	(0.85)	(0.59)	(0.85)	(0.59)
CRT01	$$0.76^{**}$$		$$0.72^{*}$$	
	(0.38)		(0.37)	
STRAT:CRT01	$$-0.97^{**}$$		$$-1.04^{**}$$	
	(0.48)		(0.47)	
CRT.DUMMY		$$0.48^{**}$$		$$0.47^{**}$$
		(0.24)		(0.23)
STRAT:CRT.DUMMY		$$-0.64^{**}$$		$$-0.69^{**}$$
		(0.27)		(0.27)
Gender			$-0.19$	$-0.22$
			(0.17)	(0.17)
Age			$$-0.02^{***}$$	$$-0.02^{***}$$
			(0.01)	(0.01)
Training question			0.19	0.13
			(0.30)	(0.30)
AIC	230.96	230.04	213.08	211.35
BIC	243.62	242.71	232.81	231.07
Log Likelihood	− 110.48	− 110.02	− 98.54	− 97.67
Deviance	58.59	58.02	49.08	48.11
Num. obs.	93	93	87	87
Power analysis				
df-num	3	3	6	6
df-den	89	89	80	80
Effect size	0.05	0.06	0.18	0.20
Significance	0.05	0.05	0.05	0.05
Power	0.4	0.48	0.83	0.88

The dependent variable is the outcome in the Distribution game that measures pro sociality. The term strat is a dummy variable taking value one for subjects with a guess lower than 50 in the GG. The term CRT01 is a dummy taking value 1 for subjects responding correctly to at least one question in the CRT and zero otherwise. CRT.DUMMY is a dummy variable taking value 1 for subjects with no correct answers to the CRT, value 2 for those responding correctly to 1 or 1 questions, and 3 otherwise. The term *gender* is equal to 1 for male and *training question* takes value 1 for subjects responding correctly to the training question of the GG. Significance Levels: $$^{***}p<0.01$$, $$^{**}p<0.05$$, $$^*p<0.1$$.

It is worth mentioning that in our regressions the variable age is always strongly significant: in our sample, young people are more prosocial, as it is evident from Models 3 in Table 6 (and Table S7). Age is particularly important, given its established negative relationship with cognitive abilities. In our setup, no statistically relevant link between this variable and strategic thinking or reflectivity has been found (Table S27) pointing to the fact that the relationship between and pro sociality is a direct one.

When estimating linear models, the presence of multicollinearity might partially depend on the inclusion of an interaction term. However, here we are interested to the interacting effect of two variables that measure different cognitive abilities which nonetheless could be reasonably correlated. We calculate the variance inflation factor for the models considered and verify that it assumes moderate values, supporting the validity of our results (Table S10). To add further robustness to our choice of variables we run a stepwise regression. While this approach is not without his own drawbacks (as it can lead to biases in the estimation, and in particular to increased Type I errors associated with inflated F values^67,68^) we use it for robustness and we report the results in Fig. S1 of the SI. The results suggest that the best model requires the use of the interaction term between CRT and GG results, in line with what we do in our main analysis (Eq. 1). An interaction plot analysis (reported in Sect. S1.1.2 of SI) confirms the importance of the interaction term. Given these reassurances, while we are aware of the higher variance in the estimation of the coefficient, we decide to keep our strategy.

A second important issue to stress is that the results of an estimation including interacting terms with dummy variables implies that the effect of one regressor on the dependent variable changes according to the different values assumed by the other regressor. Thus, to understand the effect of strategic reasoning and reflectivity on pro sociality, it is necessary to evaluate the different subsamples of people that can be classified according to the two classifications. The effect of strategic reasoning on pro sociality for subjects which are impulsive is different from its effect for those that are reflective. Similarly, to understand the effect of reflectivity on pro sociality, it is necessary to look at the different subsamples of people classified according to the level of strategic reasoning. This is done in Table 7 where *theoretical coefficients* indicate how to classify types of participants according to the theoretical estimation of the equation indicated on top of the same table (Eq. 1). Once the regression has been estimated at the individual level to preserve the heterogeneity deriving from the values of their individual covariates, the estimated coefficients must be substituted in the theoretical table to obtain the average *calculated coefficients* at the group level, reported at the bottom of the table. The robustness check with the CRT.01 specification is reported in Table S12, with similar results.

**Table 7:** Theoretical and estimated coefficients: Model 2, Model 4.

Theoretical coefficients				
Equation:				
$$PROSOC (DG)=\alpha + b_1\,STRAT +b_2\,CRT.DUMMY+b_3\,STRAT:CRT.DUMMY$$				
	naïve (strat=0)		strategic (strat=1)	
intuitive (crt.dummy=0)	$\alpha$		$\alpha +b_1$	
middle (crt.dummy=1)	$\alpha +b_2$		$\alpha +b_1+b_2+b_3$	
reflective (crt.dummy=2)	$\alpha +2b_2$		$\alpha +b_1+2b_2+2b_3$	
Calculated coefficients				
	Model 2		Model 4	
	naïve	strategic	naïve	strategic
intuitive	1.43	2.72	2.34	3.60
middle	1.92	2.57	2.81	3.39
reflective	2.40	2.42	3.29	3.17

Theoretical coefficients indicate how to calculate the coefficients related to each type of subject according to the two classifications (Strategic-reflective), using the estimated coefficients from Equation (1) and reported in Table 6. To obtain the exact values of the coefficients of the table, both calculated and theoretical, it is necessary to substitute the values of strat (0,1) and crt.dummy (0,1,2) in the equation at the top of the table, to obtain the resulting composed coefficients presented later in the Table, and using the estimated values of the regression of Table 6.

We notice that the coefficients that measure the link between strategic reasoning and pro sociality in Table 7, are always higher than those of non strategic subjects: this result is robust to all presented specifications, which include two classifications for the CRT output, the inclusion of the control variables and the use of the full or cleaned dataset. As a further robustness check, we confirm our results reporting the estimated marginal means from the regressions discussed (Table 8), as well as the interaction-style plots (Fig. 4). Overall, these estimations allow us to state our first result:

**Table 8:** Estimated marginal means for DG.

	naïve	strategic
	(strat=0)	(strat=1)
intuitive (crt.dummy=0)	1.86	2.56
middle(crt.dummy=1)	2.5	2.42
reflective (crt.dummy=2)	2.8	2.26

### Result 1

Strategic reasoning is positively related to pro sociality.

**Figure 3:**
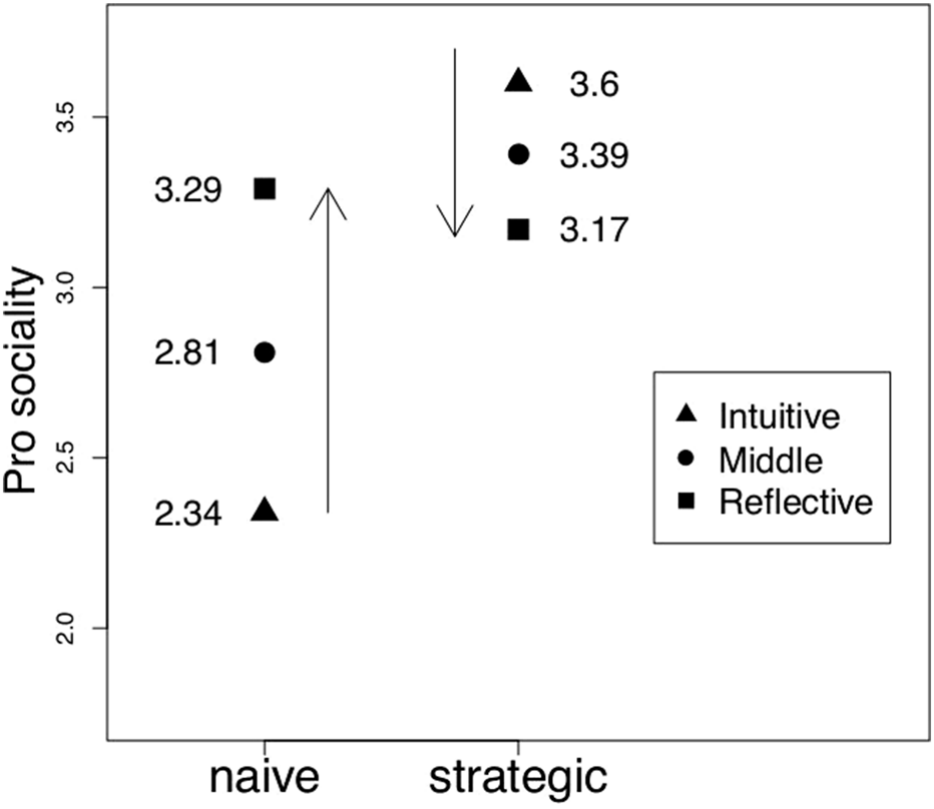
Calculated Coefficients of Model 4 (Table 7).

Figure 3 summarizes graphically the three-way relationship existing among reflectivity, strategic reasoning and pro sociality resulting from our estimated Model 4, while results are in Tables 7 and S12. We only discuss Model 2 and 4 in detail because they are the most complete and report best performance in terms of AIC, BIC and power value (83% and 88% respectively). We are aware of the controversial use of power significance in a post-analysis comparison^69^, but we report it nonetheless given that all models are significant, the significance level has been fixed to 95% a priori and sample size is constant across models and, as discussed, this is not the only criterion used to make our choice.

As indicated by the arrow, the level of pro sociality is higher for reflective subjects than for intuitive subjects when naïve subjects are considered (Fig. 3). On the contrary, when strategic subjects are considered, the arrow points downward: more reflective subjects are on average less prosocial. This evidence, qualitatively robust to all the aforementioned specifications, suggests our second result:

### Result 2

Reflectivity relates to higher pro sociality among naïve subjects and to lower pro sociality among strategic subjects.

Our results together suggest that while being strategic per sé leads to a higher tendency to be prosocial, reflectivity has a different impact on pro sociality, depending on whether subjects are capable of strategic sophistication or not.

**Figure 4:**
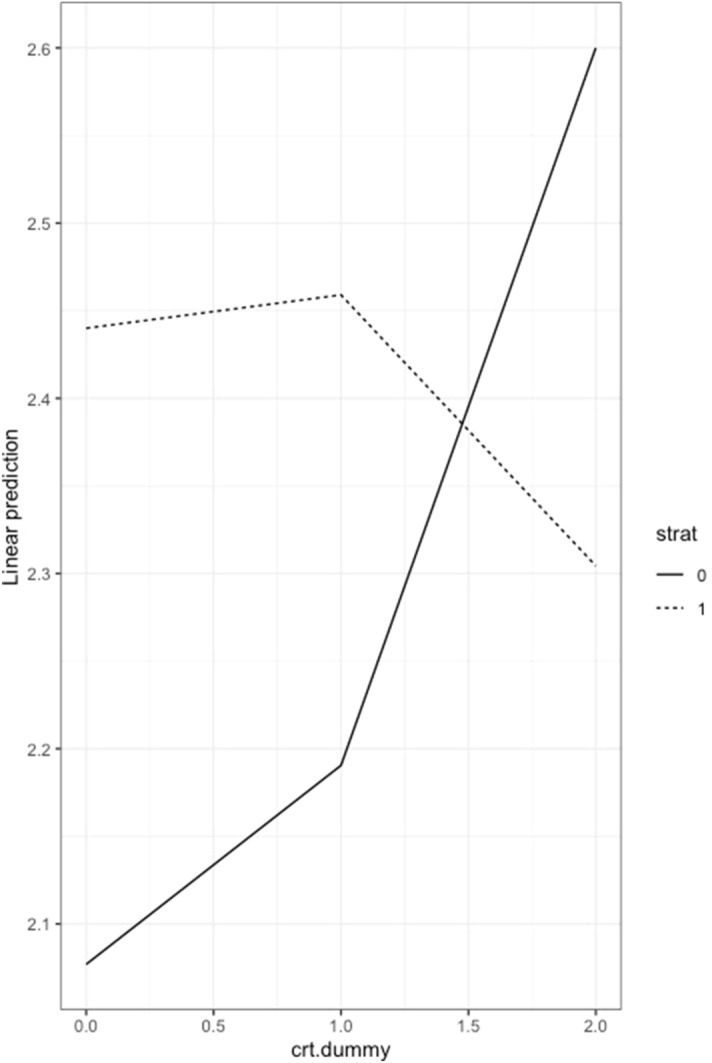
Interaction-style plot for estimated marginal means.

We note the fact that our results emerge more neatly when we consider the dataset excluding answers from subjects that explicitly admitted to have hazarded a guess in that task. This suggests that the information provided by the written comments of the participants qualify their choices in the Guessing game. Written accounts constitute a significant improvement in the understanding of the role of the guessing game as a measure of strategic reasoning because they allow to remove spurious choices coming from subjects who admitted having responded at random and at the same time providing a guess belonging to the range of strategic guesses. It can also explain the lack of a significant relationship between strategic reasoning and pro sociality found in previous contributions studying the relationship between strategic reasoning and pro sociality^44^. While written accounts are not incentivized and may also be in principle prone to false answers, there are no incentives for a participant to falsely declare to have guessed an answer. As a robustness check we also run our complete set of analyses on the full dataset (where random guesses are not dropped) qualitatively confirming both results. Results are reported in Sect. S1 of SI.

One potential alternative explanation for our results is that subjects engaging in higher levels of reasoning in the Guessing game might be more cognitively depleted, which in turn, could have led to more intuitive responses in the CRT (played at the end of the experiment). While this alternative cannot be fully discounted with our experimental setup, we consider it as less likely for several reasons. First, we find no evidence in the literature^32–35,70^ that high reasoning in the GG causes significant depletion in the successive games, while Peysakhovich  & Rand^13^—similarly to us—use a CRT played after a long battery of strategic and not strategic games, to classify individuals according to their reflectivity. Second, the one-shot guessing game is a relatively fast task that is unlikely to cause significant depletion. Finally, the elimination of random guesses mitigates the risk of uneven levels of cognitive depletion among subjects. A complete disambiguation between these alternative explanations deserves further investigation, possibly with complementary experimental strategies, including treatments that shuffle game order.

## Discussion

Whether humans are naturally good to one another or are rather evolved to fight one against the other for survival, is a fascinating question. Indeed, social evolution, culture and education taught humans to control their natural dispositions. But which are the natural dispositions and which are the learned behaviors is subject to a lively debate.

In our paper, we explore this issue experimentally and find two interesting results. Our first result states that being pro social is positively related to strategizing. This result suggests that the propensity to make decisions that favor others at a cost to oneself and considering others’ potential behaviors when taking a decision, seem to belong to a common cognitive root which allows a person to understand the relationship between his decisions, the decisions of the others, and the outcome that follows from that, and at the same time to perceive that his own behavior can affect others.

We can venture an explanation to our results through empathy, which is the natural response to individual perception of others’ feelings^51^. Empathy is a process that is activated in some humans even in absence of any direct emotional stimulation: thus promoting other-regarding behavior as an automatic process^51^. Singer, T. & Fehr, E.^51^suggest that there exists a connection between higher empathic abilities and better faculty to predict other’s motives and actions, i.e. strategic thinking. Moreover, given that empathy is heterogeneous across individuals^71,72^, people with stronger empathic abilities can be identified as those predicting better others’ motives and actions. Our result could then be explained as an empathic automatic response against unfair allocations of income, internalized by subjects with cognitive abilities sharing common roots with cognitive processes characterizing strategic reasoning^53^. In support of this speculation, recently Schurz et al.^53^ mapped tasks belonging to strategic reasoning (or Theory of Mind, ToM) and empathy to the set of underlying processes linked to concrete experimental tasks, in search of the common neurocognitive components that they involve. This exercise, support the idea that *empathy and ToM share specific processes and therefore brain activities.* On this regard, it is important to stress that empathy is considered as an umbrella term involving both affective and cognitive empathy. Affective empathy involves the sharing of others’ feelings, emotions or—in general—the sensory states of another person related to the activation of limbic regions which are often elicited through visualization of strong emotions (in particular pain). This process is thought to generate isomorphic affective states by direct signaling^73^. Contrastingly, cognitive empathy involves the cognitive inference on another person’s affective state. This is a process of perspective taking over others’ states, which does not require the direct experience of emotions, but nonetheless involves both perspectives taking on others and the identification of others’ feelings, often through simulation of own states^73^. Cognitive empathy is associated with both processes and brain regions associated with mentalizing as well as with those involved in affective processing (i.e. it shows similarities to the processes involved in the direct experience of emotions). Thus, the cognitive component of empathy is the one that can be linked to pro sociality, in line with^74^, which found that similar brain regions that are involved in both the mentalizing network and the empathy network are also linked to pro sociality.

Our second result indicates that the group of subjects capable to understand that their actions may affect the others (strategic subjects) are, on average, intuitively prosocial. For these individuals, being selfish requires deliberation, so that being more reflective acts as an anti-social device, consistently with the intuitive pro sociality view^75^. While this attitude characterizes strategic subjects, they do not always exercise it: in line with^14^, strategic subjects are on average innately pro social but can exert selfish behavior with reflectivity. Accordingly, we observe a difference between the natural-intuitive response and the reflected choice, consistently with the principle of a dual process of reasoning^76^. In turn, the group of non-strategic subjects is intuitively more selfish. They can be prosocial but they need to make an effort, they need to reflect on it: their nature does not lead them to perceive instinctively that their actions may influence others, so they naturally exercise their selfish attitude when acting impulsively. While a tight empirical test for this explanation is beyond the reach of the experimental design described in this manuscript, this hypothesis deserves being studied in further research.

Naturally, our work has limitations. From the theoretical viewpoint what presented here is only a first step to attempt a disentanglement of the composite effects of strategic reasoning and reflectivity on pro sociality. Indeed, these cognitive elements have both commonalities and differences which need further dedicated designs to be fully disentangled. Furthermore, the DG is only one of the possible measures of pro sociality, and different measures could possibly lead to different results. Additionally, further investigation with complementary experimental strategies, shuffling the order of tasks, is needed to completely rule out the possibly of heterogenous levels of depletion. Finally, it is important to stress that our results are obtained with a sample of the general population in a region of Northern Italy, instead of a standard undergraduate students sample. Beside limiting the achievable sample size, it should be noted that according to the Social Heuristics Hypothesis, the intuitive mode of pro sociality is the result of experience, culture and education, implying that results could be different in other regions in Italy and even more in other countries. Future research will involve an investigation of these possible heterogeneities.

## Methods

### Procedures

The experiment was conducted using the Reggio Emilia Behavioral and Experimental mobile Laboratory (REBEL) of the University of Modena and Reggio Emilia, using last generation tablets. Experimental sessions were run in the Italian provinces of Modena (in the municipalities of Vignola and Mirandola) and Reggio Emilia (in the main town of the province) between November 2015 and May 2016. In Vignola and Mirandola the experimental sessions were organized in the municipalities’ council chambers while in Reggio Emilia, the experimental sessions were organized in a large meeting room at the university. All venues used for the experiment were near or at well-known locations. They were all accessible by car and public transport. Socio-demographic informations about the municipalities of the three locations are reported in Table S28.

For each of the three locations, individuals were recruited from the general population through a procedure aimed at maximizing the diversity in the sample. First, 100 letters were sent to a group of families randomly extracted from the population of the municipalities involved. Then, flyers and posters advertising the event were affixed in a large number of public places (such as bars, restaurants and shops). The events were advertised through the municipalities newsletters and through a Facebook page. Finally a large ($\sim 4000$) number of ex-students (graduated from 2009 to 2015) from all faculties of the University of Modena and Reggio Emilia were contacted through email, inviting them to spread the information about the experiment. Interested individuals were invited to contact the researchers by email, through a web-form on a publicly available webpage, or by phone, and were then randomly assigned to an experimental session organized in their municipality of residence or nearby.

The selection of the participants from the pool of candidates was made imposing four restrictions. First, subjects had to be 18 years or older. Second, they had to be current residents in the municipality where the experimental sessions were run, or in the neighboring municipalities. Third, subject were asked to declare that they had no open criminal charges. Finally, running the experiment in towns where no experimental lab exists, we are confident that the vast majority of the participants never previously participated in behavioural experiments. The fact of having individuals naïve to the experimental settings puts us in line with results from both ISH^15^ and SHH^6^ which find relationship between pro sociality and intuition for this type of participants.

The whole experiment was developed in Python on the o-Tree web-based platform^77^. This software platform has been developed for running experiments on touchscreen based mobile/tablet workstations with easy of use visual interfaces. This facilitates the understanding of the software across a population not necessarily possessing computer proficiency. The easiness of use of both o-Tree and tablets was assessed by the participants in a very positive way (see Table S29 of SI).

Upon arrival at the experimental session, subjects were registered and assigned a seat. Participants were given both an informed consent as well as a privacy consent and data release form to read and sign. Participants were informed they could leave at any moment, but nobody did. During the experimental sessions visual contact among participants was made impossible by the use of mobile cubicles, furthermore all participants were informed of the fact that oral communication was forbidden.

During the whole experiment the relevant instructions appearing on the tablets were read aloud by one experimenter (always the same for all sessions). The relevant instructions were available at the bottom of the screen at any time during the task and freely accessible by the participants.

The experiment was conducted in accordance with regulations and relevant guidelines for experiments with human subjects of the REBEL (Reggio Emilia Behavioural Economics Laboratory) at the University of Modena and Reggio Emilia and therefore approved by the REBEL’s ethics committee.

Average session time was one hour, and payoffs were expressed in experimental points (tokens), with each token corresponding to 0.04 €. The average payment per person was around 15 €. The composition of our final sample is outlined in Table 9 and detailed in Table S29. Our recruitment strategy allowed us to gather a sample of individuals significantly different from a student-only one. Indeed, while our sample overweights students, young individuals and females, the whole spectrum of the local population is represented in the sample both in terms of age, sex, and working status.

The number of participants was 16 for each experimental session, with every individual allowed to participate in only one session. All sessions were run on Saturdays in order to favor a wider and more diverse participation.

**Table 9:** Socio-demographic data of the experimental sample.

Participants: N =176					
Age classes:		Gender		Work status:	
18-25	73 (41,5%)	Male:	61 (34%)	Employed	82 (46,6%)
26-35	36 (20,4%)	Female:	112 (64%)	Not Active*	25 (14,2%)
36-45	17 (9,7%)	NA	3 (2%)	Student	67 (38,1%)
46-55	20 (11,4%)			NA	2 (1,1%)
56-65	16 (9,1%)				
65+	8 (4,5%)				
NA	6 (3,4%)				

Percentages are out of the total number of participants, N=176. *Not Active refers to: housewives, retired, unemployed and unoccupied individuals.

## Supplementary Information

Supplementary Information 1.
