# Supplementary material for: Reflectivity relates differently to pro sociality in naïve and strategic subjects
Source: Sci Rep. 2021 Jun 17;11:12745. doi: 10.1038/s41598-021-91960-3 (PMC8211810; doi:10.1038/s41598-021-91960-3)
Supplement: Supplementary file 1 — Supplementary Information 1. [file 41598_2021_91960_MOESM1_ESM.pdf]

Reflectivity relates differently to pro sociality in naïve and strategic subjects
Supplementary Information

Francesca Pancotto[1] and Simone Righi[2]

[1]University of Modena and Reggio Emilia, Viale Allegri 9, 42121, Reggio Emilia (Italy). francesca.pancotto@unimore.it
[2]Ca' Foscari University of Venice, Department of economics, Fondamenta S. Giobbe, 873, 30121 Venezia (Italy). simone.righi@unive.it

Contents

S1 Comparative analysis between DG game and the PGG (run on the full dataset) 3
S1.1 Statistical analysis 4
S1.1.1 Stepwise regression 4
S1.1.2 Interaction plots 7
S1.1.3 Regression analysis 9
S1.1.4 Calculated coefficients 13
S1.1.5 A comment to the comparative analysis 14

S2 Estimated marginal means 16
S2.1 Comparing effect size between models with complete and reduced datasets . 18
S2.2 Variance Inflation Factors 18

S3 Discussion of exclusion criteria for random choices in the GG 19

S4 Literature review to support the potential connection between mentalizing, empathy and pro sociality 22

S5 Additional Statistical Analysis 25

S6 Structure of the Sample and Experimental Localities 38

S7 Translation of the Experiments 40

S8 Choice screens vignettes 56

S9 Extended CRT questions from Primi et al. 2016 56

S10Parameters of the Distribution Game 58

List of Figures

S1 Stepwise procedure with interaction term: DG 5
S2 Stepwise procedure with interaction term: PGG 6
S3 Interaction plot for PGG (crt.dummy variable) 8
S4 Interaction plot for DG (crt.dummy variable) 8
S5 Interaction plot for PGG (CRT variable) 8
S6 Interaction plot for DG (CRTvariable) 8
S7 Interaction-style plots for estimated marginal means 17
S8 Top: histogram of choices in the guessing game, counts and density. Bottom: Density distribution of choices comparing complete database and clean (excluding random guesses). 22
S9 Estimated Coefficients Model 3 (of Table S12) 27
S10 Guessing game choice screen 56
S11 Dictator game choice screen 56

List of Tables

S1 Pro sociality measured with Distribution Game (DG)(Stepwise regression model) 11
S2 Pro sociality measured with Distribution Game (DG) 11
S3 Public Goods Game (PGG) 12
S4 Public Goods Game (PGG) (stepwise regression model) 12
S5 Theoretical and estimated coefficients: DG and PGG 14
S6 Estimated coefficients:PGG 14
S7 Estimated marginal means: complete and reduced dataset 17
S8 Estimated marginal means analysis for the DG (complete dataset) 17
S9 Pro sociality, strategic reasoning and reflectivity. Complete and reduced dataset. Comparing effect size 18
S10 Variance inflation factor: Complete and reduced dataset. Models including and excluding interactions. 19
S11 Pro sociality, strategic reasoning and reflectivity. OLS with standard errors clustered at session Level 26
S12 Theoretical and estimated coefficients: Models 1 and 3 27
S13 Pro sociality. OLS. Dataset including random guesses in GG 28
S14 Dependent variable: Pro sociality. OLS 29
S15 Dependent variable: Pro sociality. Clustered standard errors 30
S16 Dependent variable: Pro sociality. Ordered logit 31
S17 Ordered Logit. Odd ratios and Confidence Intervals. Model 1 31
S18 Ordered Logit. Odd ratios and Confidence Intervals. Model 2 32

S19 Ordered Logit. Odd ratios and Confidence Intervals. Model 3 32
S20 Ordered Logit. Odd ratios and Confidence Intervals. Model 4 32
S21 Ordered Logit. Odd ratios and Confidence Intervals. Model 5 33
S22 Ordered Logit. Odd ratios and Confidence Intervals. Model 6 33
S23 Pro sociality. Ols with dependent variable DG codified in two categories . . 34
S24 Pro sociality. Ols with dependent variable DG codified in two categories. OLS with standard errors clustered at session level 35
S25 Pro sociality. Logit with dependent variable DG codified in two categories . 36
S26 Pro sociality. Logit model with standard errors clustered at session level . . 37
S27 Linear regression of age on guess, crt and dictator 37
S28 Characteristics of the Municipalities involved in the experiment 38
S29 Structure of the Sample 39
S30 Parameters of the Distribution Game 58

S1 Comparative analysis between DG game and the PGG (run on the full dataset)

The experiment described in this paper consisted of a series of tasks, on a sample of the general population completed in the order presented in Table 1, main text. The first task is a Public Goods Game. We briefly describe the main feature of the Public Goods Game that we use as a robustness check on the results of our paper.

The Public Goods Game We use a discrete form Public Goods Game, in order to make the choice easier for participants: all group members receive an endowment of $e = 40$ experimental points and have to decide simultaneously how much of their endowment to invest in a common project, choosing a contribution level among the following possible values, ($c_i \in 0, 10, 20, 30, 40$), knowing that the residual from this contribution would remain in their private account. Every point invested in the group is doubled and shared equally among group members. Individual earnings are determined according to the following equation, with π defined as the payoff:

$$\pi_i \;=\; e - c_i + \alpha \sum_{j=0}^{N} c_j,$$

where α is the marginal per capita return (MPCR) of the public good. Free-riding is the dominant strategy, for rational self-interested individuals, because MPCR is above 1/N and below 1. Social welfare is instead maximized when everyone contributes the whole endowment. These two opposing results define this game as a social dilemma.

S1.1 Statistical analysis

Here we report the study of the statistical relationship between pro sociality and cognitive abilities elicited with the guessing game and the CRT game, using both the results of the DG game and the PGG game.

S1.1.1 Stepwise regression

As a preliminary procedure, we run a backward stepwise regression analysis on both models, to explore the best available model according to the data, using the *R Mass* package.

The results for DG equation are reported in Figure S1. The stepwise regression suggests to use the results of the CRT and of the guessing game (GUESS), the interaction variable between the two and whether the indivduals answered correctly to the comprehension questions made before the game (gg_right1).

For the PGG equation, the results of stepwise regression are reported in Figure S2 and support the inclusion of the interaction term and of the age of participants, but not of the main effects of strategic thinking and reflectivity. We will test the relevance of the interaction for the PGG later in the analysis.

Figure S1: Stepwise procedure with interaction term: DG

```
Start:  AIC=-61.19
dictator ~ guess + gender + age + crt + gg_right1 + interaction

              Df Sum of Sq    RSS     AIC
- gender       1   0.22608 106.69 -62.832
- age          1   0.68665 107.15 -62.113
<none>                     106.46 -61.186
- gg_right1    1   1.29275 107.75 -61.171
- crt          1   1.30192 107.76 -61.156
- interaction  1   2.42464 108.89 -59.426
- guess        1   2.47849 108.94 -59.343

Step:  AIC=-62.83
dictator ~ guess + age + crt + gg_right1 + interaction

              Df Sum of Sq    RSS     AIC
- age          1    0.7371 107.42 -63.682
<none>                     106.69 -62.832
- gg_right1    1    1.3657 108.05 -62.708
- crt          1    1.5825 108.27 -62.373
- interaction  1    2.4443 109.13 -61.049
- guess        1    2.5598 109.25 -60.872

Step:  AIC=-63.68
dictator ~ guess + crt + gg_right1 + interaction

              Df Sum of Sq    RSS     AIC
<none>                     107.42 -63.682
- crt          1    1.3516 108.78 -63.594
- gg_right1    1    1.6916 109.11 -63.073
- interaction  1    2.1431 109.57 -62.383
- guess        1    2.5587 109.98 -61.751
```

Figure S2: Stepwise procedure with interaction term: PGG

```
Start:  AIC=831.88
pgg ~ guess + gender + age + crt + gg_right1 + interaction

              Df Sum of Sq   RSS    AIC
- crt          1     18.44 22389 830.02
- interaction  1     18.80 22389 830.02
- gender       1     85.82 22456 830.52
- guess        1    116.44 22487 830.75
- gg_right1    1    161.82 22532 831.08
<none>                     22370 831.88
- age          1    928.56 23299 836.67

Step:  AIC=830.02
pgg ~ guess + gender + age + gg_right1 + interaction

              Df Sum of Sq   RSS    AIC
- gender       1     73.81 22462 828.57
- guess        1    108.60 22497 828.83
- gg_right1    1    159.67 22548 829.21
- interaction  1    219.68 22608 829.65
<none>                     22389 830.02
- age          1    910.23 23299 834.67

Step:  AIC=828.57
pgg ~ guess + age + gg_right1 + interaction

              Df Sum of Sq   RSS    AIC
- guess        1    127.81 22590 827.52
- gg_right1    1    147.63 22610 827.66
- interaction  1    166.26 22629 827.80
<none>                     22462 828.57
- age          1    893.16 23356 833.08

Step:  AIC=827.52
pgg ~ age + gg_right1 + interaction

              Df Sum of Sq   RSS    AIC
- gg_right1    1    188.37 22779 826.90
<none>                     22590 827.52
- interaction  1    353.86 22944 828.11
- age          1    971.47 23562 832.55

Step:  AIC=826.9
pgg ~ age + interaction

              Df Sum of Sq   RSS    AIC
<none>                     22779 826.90
- interaction  1    323.49 23102 827.26
- age          1   1137.25 23916 833.04

Call:
lm(formula = pgg ~ age + interaction, data = tmp4)

Coefficients:
(Intercept)          age  interaction
   18.08247      0.17430      0.01679
```

S1.1.2 Interaction plots

In this paragraph we report the comparative interaction plot, aimed at measuring whether the interaction term, suggested by the step wise procedure, should be included in the estimation of our model for the DG and the one for the PGG.

An interaction plot is a simple line graph examining interactions between variables in factorial designs. Quoting (Lovie, 2014) on how to read interaction plots "*For a two factor design, the cell means on the response variable for each level of one factor are plotted over all the levels of the second. The resulting profiles are parallel when there is no interaction and nonparallel when the interaction is present*". In our case, we are interested to the effect on two response variables, the choices in PGG and DG, of two factors, i.e. strategic reasoning and cognitive reflection at the center of the analysis reported in the main text. Our interaction plots report then on the x-axis, the dichotomous variable STRAT, on the y-axis the mean value of the dependent variables (PGG and DG), and then the lines represent values of CRT and CRT.DUMMY variables.

Fig. S3 shows the interaction plot for PGG. For the PGG, the values of CRT for value of STRAT=1 are always lower than for the value STRAT=0. The higher the level of strategic reasoning, the lower contribution, independently from the value of CRT.DUMMY, although this effect is nonetheless stronger for people showing higher reflectivity. In this plot we do not see crossing lines, which suggests that - for this game - no interaction can be observed between the two dimensions. Fig. S4 shows the interaction plot for the DG. In the interaction plot of the DG, the lines crossing show that the interaction is an important variable in this case. Accordingly we have included it in our main analyses. Moreover, high values of CRT (CRT.DUMMY=1,2) have a positive slope while low values have a negative slope. With similar results, the interaction plots created from the CRT raw results are reported in Fig. S5 for the PGG and Fig. S6 for DG.

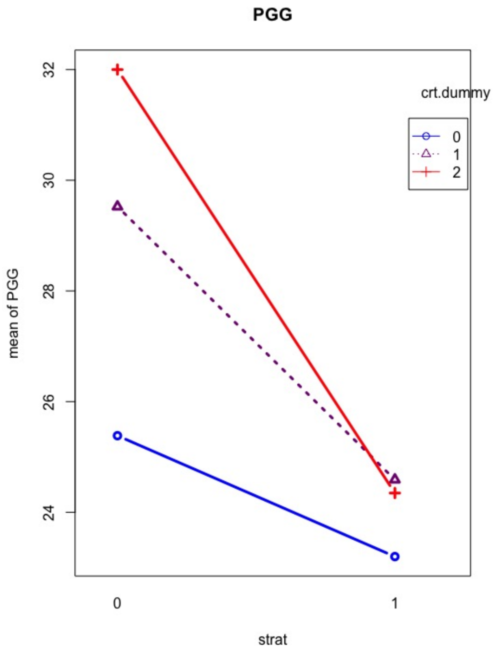

Figure S3: Interaction plot for PGG (crt.dummy variable)

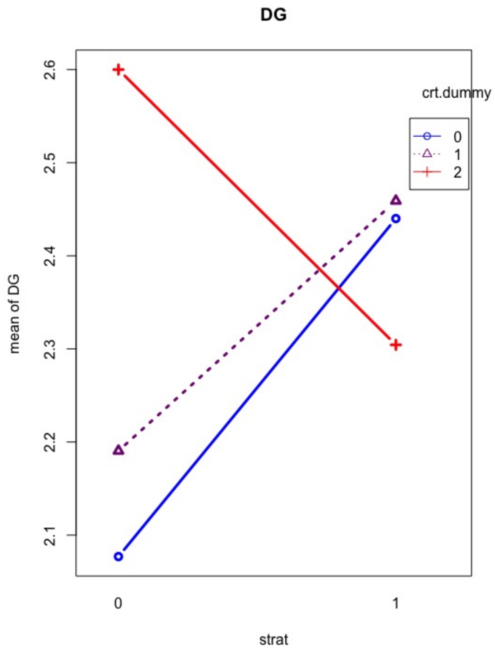

Figure S4: Interaction plot for DG (crt.dummy variable)

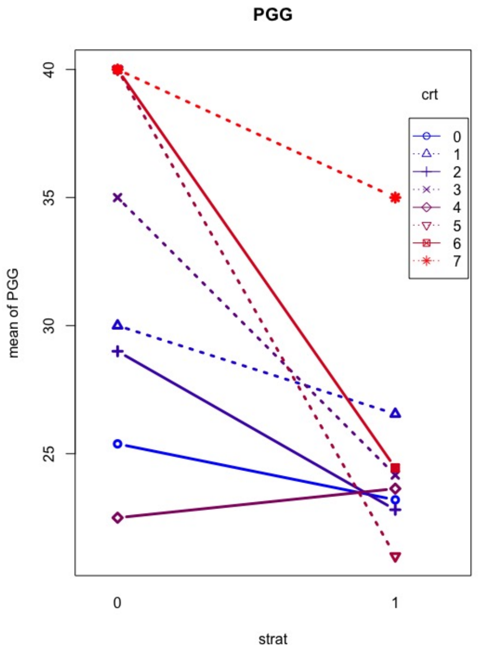

Figure S5: Interaction plot for PGG (CRT variable)

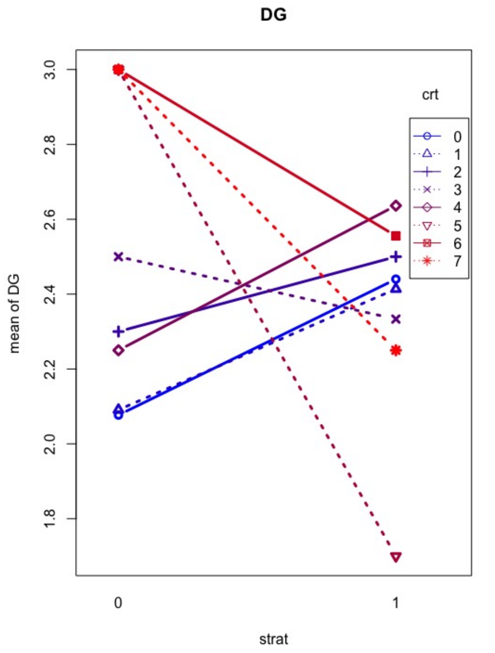

Figure S6: Interaction plot for DG (CRTvariable)

S1.1.3 Regression analysis

The final step of our analysis include the comparison of a regression analysis using as dependent variable both the choices in PGG and in DG respectively, including the data for the whole sample (i.e. without eliminating people declaring random guesses). This analysis provides a test of the appropriateness of the using the DG as a tool to test our hypothesis and excluding instead the PGG. We also provide a robustness check to the result on the DG reported in the main text, where data of random guesses in the guessing game have been dropped. The procedure adopted is the same as the one used in the main text, but here we are running regressions with the complete dataset ($N = 176$), thus providing a robustness check for our results, and confirming that they are stable to the use of both datasets.

In Table S2, we study the model for the DG: Model 1 reports the model with variables GUESS (the raw choices in the guessing game) CRT (the number of correct answers in the CRT), and the interaction term between the two. We select this model according to the result of the stepwise regression analysis in Section S1.1.1 and in analogy with what presented in the main text. Model 2, replicates the analysis using the STRAT variable, while Model 3 uses both dummy variables STRAT and CRT.DUMMY. Overall the models cover all the different possible specifications of the variables, i.e. raw data (variables CRT, GUESS) and dummy variables of the same data (variables CRT.DUMMY, STRAT) which allow to read results more clearly.

We find that the variable related to the guessing game is always significant, both as raw variable, and as dummy STRAT. In the first case, the coefficient is negative (Model 1 Table S2), which means that larger guesses are related to lower fairness (pro sociality in the DG can be called fairness, to distinguish it from pro sociality measured as cooperation in the PGG): less(more) strategic subjects are less(more) prosocial if we only consider this term. In Model 1, also the CRT variable is significant and negative, which could indicate that reflectivity leads to lower pro sociality. These conclusions need to be complemented by the consideration of the value and of the sign of the interaction term. The interaction term is of opposite sign with respect to both coefficients, indicating that the interaction could change the effect of the direct coefficients on the dependent variable. To make a conclusion it is thus necessary to measure the net effect. The value of the coefficient is also very small because of the different scale of the two variables: to calculate with higher clarity this effect, we perform this analysis using the dummy versions of the variables.

In Model 2, the coefficient of dummy STRAT, is significant and positive: the dummy STRAT is codified to have value 1 for subjects with higher levels of strategic reasoning , which correspond to values of guess lower than 50. This explains the inversion of the sign of the coefficient of Model 2 with respect to Model 1, but the information reported is consistent. Thus the positive value shows that strategic subjects are more pro social (or fair). CRT variable here is also significant, but the coefficient changes sign, due to the change in codification of the variable guess. Finally, the interaction term is significant and with negative sign, which imposes us to calculate the net effects of coefficients in order to evaluate the influence of regressors on dependent variable. Model 3 shows the results using all dummy variables, which simplify the calculation of net effects.

In Table S1, we extend the models reported in Table S2, to include the variable *training_question* which is the variable capturing whether subjects have answered correctly to the control question for the guessing game. This extension of the model is suggested by the stepwise regression analysis, which always considers this variable in the final step of the procedure. For remaining variables, results do not change qualitatively with respect to the models in Table S2. Considering that models including *training_question* always present better statistics related to the quality of the regression, we use these for the analysis of calculated coefficients. We also stress that the results are consistent across the three Models presented, so we use Model 3.

In Table S3 we run regressions using PGG as dependent variable. The results of the stepwise regression did not suggest to use an interaction term, however we tested the model for comparison with the DG and found no significance. In Model 2, we eliminate the interaction and just tested the correlation between direct effects of crt and guess on the dipendent variable: here we find that a higher guess in the Guessing Game is positively correlated with the choice in the PGG. We also test the models with dummies, and the results confirm that for the case of the PGG, the only significant variable is STRAT, negatively related to pro sociality. Table S4 reports the estimates using the model proposed by the stepwise, i.e using the guess and the variable age. Here the effect on the choice in the PGG is basically related only to the level of strategic reasoning and age.

Table S1: Pro sociality measured with Distribution Game (DG)(Stepwise regression model)

	Model 1	Model 2	Model 3
(Intercept)	2.46***	1.79***	1.52***
	(0.25)	(0.22)	(0.38)
guess	−0.01**		
	(0.00)		
crt	−0.11*	0.13*	
	(0.06)	(0.07)	
training_question	0.28	0.30	0.32*
	(0.19)	(0.19)	(0.19)
guess:crt	0.003**		
	(0.001)		
strat		0.43**	0.83**
		(0.20)	(0.42)
strat:crt		−0.17**	
		(0.08)	
crt.dummy			0.25
			(0.17)
strat:crt.dummy			−0.36*
			(0.20)
AIC	434.03	433.97	435.36
BIC	453.05	452.99	454.38
Log Likelihood	−211.01	−210.98	−211.68
Deviance	113.35	113.31	114.21
Num. obs.	176	176	176

Notes: Complete dataset. Guess: value of the guess in the GG. crt: number of correct answers to the CRT; STRAT: dummy equal to one for guesses in the GG smaller than 50, and zero otherwise; CRT.DUMMY: dummy taking value 1 for no correct answers,2 for two and three correct answers, 3 otherwise. Significance Levels: $^{***}p < 0.01$, $^{**}p < 0.05$, $^{*}p < 0.1$

Table S2: Pro sociality measured with Distribution Game (DG)

	Model 1	Model 2	Model 3
(Intercept)	2.71***	2.02***	1.76***
	(0.18)	(0.17)	(0.35)
guess	−0.01**		
	(0.00)		
crt	−0.10*	0.13*	
	(0.06)	(0.07)	
guess:crt	0.003**		
	(0.001)		
strat		0.45**	0.81*
		(0.20)	(0.42)
strat:crt		−0.16**	
		(0.08)	
crt.dummy			0.25
			(0.17)
strat:crt.dummy			−0.33*
			(0.20)
AIC	434.37	434.56	436.22
BIC	450.23	450.42	452.07
Log Likelihood	−212.19	−212.28	−213.11
Deviance	114.87	115.00	116.08
Num. obs.	176	176	176

Notes: Complete dataset. Guess: value of the guess in the GG. crt: number of correct answers to the CRT; strat: dummy equal to one for guesses in the GG smaller than 50, and zero otherwise; crt.dummy: dummy taking value 1 for no correct answers,2 for two and three correct answers, 3 otherwise. Significance Levels: $^{***}p < 0.01$, $^{**}p < 0.05$, $^{*}p < 0.1$

Table S3: Public Goods Game (PGG)

	Model 1	Model 2	Model 3	Model 4
(Intercept)	22.48***	21.43***	25.73***	27.76***
	(2.69)	(2.16)	(2.96)	(2.16)
guess	0.06	0.09*		
	(0.06)	(0.05)		
crt	−0.08	0.44		
	(0.92)	(0.47)		
guess:crt	0.02			
	(0.02)			
strat			−2.02	−4.89**
			(3.57)	(2.11)
crt.dummy			3.36	1.19
			(2.51)	(1.26)
strat:crt.dummy			−2.90	
			(2.91)	
AIC	1382.39	1380.83	1380.31	1379.32
BIC	1398.24	1393.51	1396.16	1392.00
Log Likelihood	−686.20	−686.42	−685.15	−685.66
Num. obs.	176	176	176	176

Notes: Complete dataset. Guess: value of the guess in the GG. crt: number of correct answers to the CRT; strat: dummy equal to one for guesses in the GG smaller than 50, and zero otherwise; crt.dummy: dummy taking value 1 for no correct answers,2 for two and three correct answers, 3 otherwise. Significance Levels: $^{***}p < 0.01$, $^{**}p < 0.05$, $^{*}p < 0.1$

Table S4: Public Goods Game (PGG) (stepwise regression model)

	Model 1	Model 2
(Intercept)	17.32***	22.70***
	(2.59)	(2.92)
guess	0.07	
	(0.04)	
age	0.16***	0.16***
	(0.06)	(0.06)
strat		−3.69*
		(2.09)
AIC	1326.43	1325.84
BIC	1338.97	1338.38
Log Likelihood	−659.21	−658.92
Deviance	23233.84	23153.71
Num. obs.	170	170

Notes: Complete dataset. Guess: value of the guess in the GG. crt: number of correct answers to the CRT; strat: dummy equal to one for guesses in the GG smaller than 50, and zero otherwise; crt.dummy: dummy taking value 1 for no correct answers,2 for two and three correct answers, 3 otherwise. Significance Levels: $^{***}p < 0.01$, $^{**}p < 0.05$, $^{*}p < 0.1$

S1.1.4 Calculated coefficients

We previously confirmed the importance of the interaction term in our regressions for DG, while the results have shown that direct and interaction terms have different signs, which implies that to evaluate the final effect we must calculate the composite coefficient.

For this analysis, we use for DG, the results of Model 3 in Table S1 and calculate the net effects using the following equation:

$$\text{PROSOC} = \alpha + b_1\,\text{STRAT} + b_2\,\text{CRT.DUMMY} + b_3\,\text{STRAT} : \text{CRT.DUMMY} + \text{GG\_RIGHT}. \quad (1)$$

To calculate the net effects, it is necessary to substitute the possible values of the dummy variables, and obtain the resulting composed coefficients. For the guessing game, we use STRAT which takes values 0 and 1, and for CRT the variable CRT.DUMMY with values 0, 1 and 2. In Table S5, we calculate composed coefficients: in the top panel, the table reports what is the coefficients for naïve subjects (STRAT=0) which made an intuitive choice in the CRT (CRT.DUMMY=0). For this subjects, the relative coefficient is α, the constant, because substituting in Equation 1 STRAT=0 and CRT.DUMMY=0, α is what is left. The calculated coefficient $\alpha + b_1 + b_2 + b_3$, is again obtained by substituting STRAT=1 and CRT.DUMMY=1 in Equation 1, and so on and so forth.

Coefficients calculated according to this procedure are reported in the bottom panel of the Table S5. The same calculations are reported in the main text for the reduced dataset, so the the results reported here on the complete dataset are a for robustness check. We find analogous results to the main text (although coefficient values are quantitatively different because of the dataset used): the coefficient that measures the effect of being at the same time strategic and reflective on pro sociality (2.13), is lower than the one that measures the effect of being strategic and intuitive (2.34) (the effect of being strategic and middle way reflective, is exactly in the middle, i.e. 2.24).

On the contrary, being naïve and intuitive, shows a coefficient of 1.51, lower than the one of subjects with naive and reflective behavior (2.01). As in the main text, reflectivity relates to lower pro sociality among strategic subjects, while it relates to higher pro sociality in naïve subjects. We can thus confirm that: STRATEGIC SUBJECTS ARE INTUITIVELY PRO SOCIAL WHILE THE OPPOSITE IS TRUE FOR NON-STRATEGIC SUBJECTS.

The results for the PGG are reported in Table S6 . Here results report only the direct effect of strategic reasoning and age on contributions, without need to resort to calculated

coefficients. According to this equation, older participants contribute more on average, while strategic subjects contribute significantly less. The absence of an interaction effect makes the calculations trivial.

Table S5: Theoretical and estimated coefficients: DG and PGG

Theoretical coefficients		
DG Equation:		
$\textsc{prosoc} = \alpha + b_1\,\textsc{strat} + b_2\,\textsc{crt.dummy} + b_3\,\textsc{strat} : \textsc{crt.dummy} + \textsc{gg\_right}$		
	naïve (STRAT=0)	strategic (STRAT=1)
intuitive (CRT.DUMMY=0)	α	$\alpha + b_1$
middle (CRT.DUMMY=1)	$\alpha + b_2$	$\alpha + b_1 + b_2 + b_3$
reflective (CRT.DUMMY=2)	$\alpha + 2b_2$	$\alpha + b_1 + 2b_2 + 2b_3$

Calculated coefficients	DG(Model 3, Table S1)	
	naïve	strategic
intuitive	1.51	2.34
middle	1.76	2.24
reflective	2.01	2.13

Table S6: Estimated coefficients:PGG

PGG Equation (Model 2, Table S4):
$\textsc{prosoc} = \alpha + b_1\,\textsc{strat} + b_2\,\textsc{age}$
$\textsc{prosoc} = 22.7 - 3.69\,\textsc{strat} + 0.16\textsc{age}$

S1.1.5 A comment to the comparative analysis

We believe that the comparative analysis qualifies in an important way our main result which is that fairness in the DG, is related to strategic reasoning, mediated by the reflectivity in the CRT.

Results for PGG and DG are different, because of the diversity of the two tasks. In the PGG, the decision to contribute depends directly from the contribution of others. Expectations of high contributions from group members, can lead to a decision to contribute a lot- in a behavioral model - or to defect- following a Nash equilibrium strategy. In all cases, subjects need to form their own expectations about the group, to decide what they want to

contribute because their payoff depends directly on the others and their expectations about them depend on their level of trust, social norm of cooperation, learnt behavior and so on and so forth.

The inner nature of the PGG game implies a strategic decision. This is also what best reply strategies or Nash equilibrium calculation procedure use to find an equilibria. For this reason, while the strategic and non-strategic games have been generally observed to produce correlated results, the PGG is not the right task to prove whether pro sociality is related to strategic reasoning, given that it is intrinsically impossible to separate strategic considerations from other motivations.

A different story applies to the distribution game that we use (DG). Participants know that they must choose an income distribution, but are informed that they their payoffs do not depend on the choices of other group members. The computer decides which state of the world that will happen after everyone made his decision. What the others do, never affects directly the individual. Each participant is has detailed information about this. In this task there is no scope for strategic considerations. The way this game is structured is crucial in determining the different results, and motivates the choice of using the distribution game as a task to measure pro sociality in our design.

S2 Estimated marginal means

We use estimated marginal means to provide a robustness check for the results presented in the main text using theoretical and calculated coefficients analysis. Moreover, this methodology allows to solve problems related to potential imbalances present in the data, estimating what the marginal means would be, had the experiment been balanced. We first calculate a reference grid using the DG as dependent variable and our dummy variables, STRAT and CRT.DUMMY and the interaction as regressors. We then use the function *emmeans* in the RPackage to calculate the estimated marginal means reported in Table S7. In the top panel we run the calculations with the complete dataset while in the bottom with the reduced dataset. The column emmean reports the calculated estimated marginal means. To compare the results of this analysis to our coefficients, we rewrite the results of Table S7 in a 3 by two matrix , which has a similar structure to the one of our analysis. This is done in Table S8. Our main results are confirmed for both complete and reduced datasets: for strategic subjects the estimated marginal means increase for increasing levels of reflectivity while for non-strategic subjects they decrease with higher reflectivity. In Figure S7a, we plot the value of the predicted marginal means for the dependent variable - choice in the DG - as a function of the value of the CRT.DUMMY, comparing strategic and non strategic subjects. The values for strategic subjects are plotted with a dashed line, while a solid line is for non strategic subjects. Strategic subjects show a predicted level of fairness that decreases with the level of reflectivity (from value 0 to value 2 of the CRT.DUMMY as well as from 1 to 2.) On the contrary, non strategic subjects, solid line, present a predicted level of fairness that increases with the level of reflectivity (positive slope of the line). In Figure S7b the same patterns appear, with a clearer effect for strategic subjects, which present lower level of predicted fairness as a function of increasing reflectivity.

Table S7: Estimated marginal means: complete and reduced dataset

Complete Dataset						
strat	crt.dummy	emmean	SE	df	lower.CL	upper.CL
0	0	2.08	0.23	170	1.63	2.53
1	0	2.44	0.16	170	2.11	2.77
0	1	2.19	0.18	170	1.84	2.55
1	1	2.46	0.11	170	2.25	2.67
0	2	2.60	0.26	170	2.09	3.11
1	2	2.30	0.12	170	2.06	2.54
Reduced Dataset						
0	0	1.86	0.31	87	1.24	2.47
1	0	2.56	0.27	87	2.02	3.10
0	1	2.50	0.29	87	1.93	3.07
1	1	2.42	0.14	87	2.14	2.71
0	2	2.80	0.36	87	2.08	3.52
1	2	2.26	0.15	87	1.97	2.55

Note: Confidence level used: 0.95

Table S8: Estimated marginal means analysis for the DG (complete dataset)

	Complete Dataset		Reduced Dataset	
	naïve (STRAT=0)	strategic (STRAT=1)	naïve (STRAT=0)	strategic (STRAT=1)
intuitive (CRT.DUMMY=0)	2.08	2.44	1.86	2.56
middle(CRT.DUMMY=1)	2.19	2.46	2.5	2.42
reflective (CRT.DUMMY=2)	2.60	2.30	2.8	2.26

Notes: Complete dataset. The values in the tables are a reorganization of the estimated marginal means of TableS7.

Figure S7: Interaction-style plots for estimated marginal means

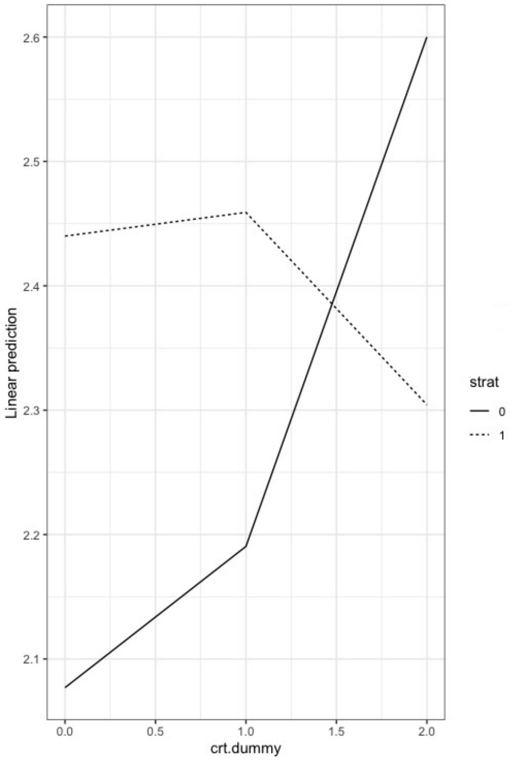

(a) Complete dataset

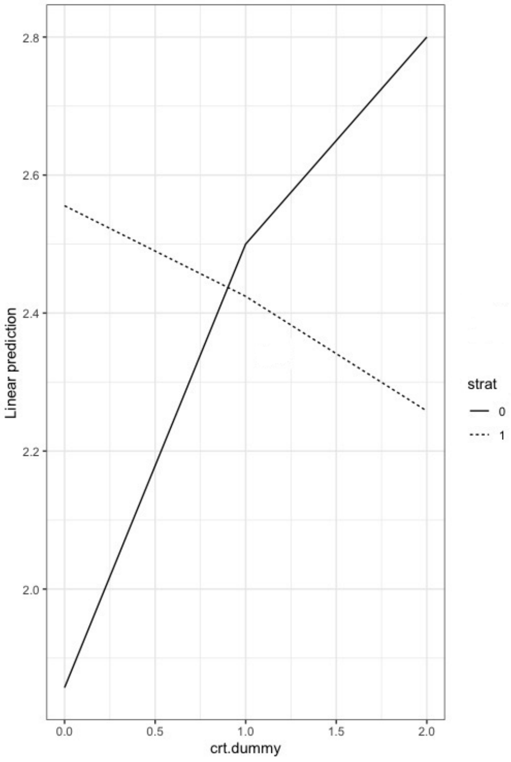

(b) Reduced dataset

S2.1 Comparing effect size between models with complete and reduced datasets

Table S9: Pro sociality, strategic reasoning and reflectivity. Complete and reduced dataset. Comparing effect size

Dep. Var. : pro sociality	Model 4 (reduced)	Model 4 (complete)
(Intercept)	2.34***	1.55***
	(0.61)	(0.48)
strat	1.26**	0.80*
	(0.59)	(0.43)
crt.dummy	0.47**	0.27
	(0.23)	(0.17)
gender	−0.22	0.06
	(0.17)	(0.14)
age	−0.02***	−0.00
	(0.01)	(0.00)
training_question	0.13	0.32
	(0.30)	(0.20)
strat:crt.dummy	−0.69**	−0.36*
	(0.27)	(0.20)
R^2	0.17	0.05
Adj. R^2	0.11	0.01
Num. obs.	87	167
RMSE	0.78	0.82
Power analysis		
df-num	6	6
df-den	80	160
effect size	0.20	0.05
significance	0.05	0.05
power	0.88	0.52

Notes: In this table we compare the estimates for the general model in the main text (Model 4 reduced) with an analogous model for the complete dataset(Model 4 complete), in order to compare power analysis. Significance Levels: $^{***}p < 0.01$, $^{**}p < 0.05$, $^{*}p < 0.1$.

S2.2 Variance Inflation Factors

As a robustness check on the use of the both the reflectivity and the strategic behaviour variables, we calculate the value of the correlation between strategizing and reflectivity, for the two datasets, which are equal to -0.26 for the reduced dataset (N=93) and -0.1 for the complete dataset (N=176).

Secondly, we report the VIF of the model estimated without the interaction term and with the interaction term and compare them. We find that the VIF increases with the inclusion of the interaction term, which is to be expected[1], but it is not so high as to create

[1] *If you specify a regression model with both x and x^2, there is a good chance that those two variables will be highly correlated. Similarly, if your model has x, z, and xz, both x and z are likely to be highly correlated with*

Nobs	Model	Guess	CRT	Age	Gender	Interaction
N=93		2.01	3.77	1.01	1.13	3.63
	w/out interaction	1.12	1.23	1.02	1.14	
N=176		2.02	4.01	1.04	1.13	4.5
	w/out interaction	1.03	1.14	1.02	1.13	

Table S10: Variance inflation factor: Complete and reduced dataset. Models including and excluding interactions.

concerns.[2] We also report for completeness, the calculation of the VIF for the model with interaction and including the complete dataset. The complete dataset presents a higher VIF for the interaction term, which suggests that our procedure to reduce data is parsimonious and in the direction of higher reliability of the data (because it reduces the potential concerns related to the correlation between terms in the interaction). Also with the complete dataset the VIF is never high enough to be cause for concern on the use of both reflectivity and strategic behaviour in our regressions.

S3 Discussion of exclusion criteria for random choices in the GG

Our experimental setup includes a series of tasks, as reported in main text, among which the non incentivized request of comments related to the Guessing Game. The procedure implemented to request these comments was the following (the detailed time line of the experiment is in Table 1, main text): first, instructions about the GG were read aloud to all participants. Participants respond to control questions and then are asked to provide their chosen number. No feedback is given to the participants concerning the GG. After the choice was made and submitted to the software, participants were asked to write on a piece of paper with ID number reported, a comment about their choice, specifying that they can explain, if they want, the reasoning process or strategy that led them make the choice in the game. Such papers were then closed in an envelope and delivered to the experimenter.

their product. This is not something to be concerned about, however, because the p-value for xz is not affected by the multicollinearity. This is easily demonstrated: you can greatly reduce the correlations by 'centering' the variables (i.e., subtracting their means) before creating the powers or the products. But the p-value for x^2 *or for xz will be exactly the same, regardless of whether or not you center. And all the results for the other variables (including the R2 but not including the lower-order terms) will be the same in either case. So the multicollinearity has no adverse consequences.* cit. Paul Allison, Multiple regression (1999)

[2]To calculate these values we used the command vif() in the R package car().

We collected these papers and codified the responses according to a basic criterium: whether participants were running consistently the Guessing Game, or whether they were using strategies unrelated to the game. In the latter case, the choices were defined as 'random' guesses.

In the following lines we list translated examples of reported comments for the GG for cases that were maintained in the database and for cases that were dropped, defined GG-random in Table 4, main text. The codification was made by three researchers independently: the results of the categorization coincided among the three.

EXAMPLES OF COMMENTS CODIFIED AS GG-RANDOM:

- The choices of the number was given randomly because I did not understand the question
- I chose 56 because I was born in 1956
- No rule, this number is my favorite number
- I chose 13 because I was born the 13th
- I chose 12 because my daughters were born on the 12th
- I chose 12 because it is the number written on the tablets I am using this morning
- I chose 38 because it is my shoe size
- I chose 31 because I dreamt this number tonight. Also, it is not too high or too low, so it seemed reasonable to me.
- It is the age of my grandma
- It is the age of a person very important to me

EXAMPLES OF COMMENTS CODIFIED AS NON-RANDOM(NOTE THAT SOME REASONING PROCESSES ARE RIGHT WHILE SOME ARE WRONG):

- Imagining that on average some people chose, 50, and some can choose around 30, I chose 22
- Paper with calculations: $50 + 18 + 20 + 80 = 168 \times 2 : 3$

- Given the possible range of values ($0 - 66 = 100 \times 4 \times 2 : 3$) and given my predictions of similar values, I fixed a range of 20-30 as winning possibilities, so I chose 25
- I thought that my choice would be 100. But as I cannot predict what the others in the group chose, I thought they may choose lower numbers, and so on I chose 90.
- If everyone chose 50 (mean number), the winning number would be close to 33. I choose 40 to make it closer to the others' choice.
- I chose thinking about what the others would choose.
- Mean of the group by 2/3. Calculations: 100+0+25+75
- I chose 20. It cannot be higher than 63 (at most). I supposed that everyone would put 30. $30 \times 2 : 3 = 20$
- I chose an average of 40 each then $40 \times 2 : 2 = 27$. So, I chose 23, which is closer, but it was my day of birth.
- I chose a not very big number in order to reduce its influence in the calculation of the general mean and in the attempt to make the values get closer to the central value of the interval.

It is important to stress that we only dropped those observations, as in the previous set of examples, where the participant appeared not to have played the game and used criteria unrelated to the game. People making a wrong statements were kept because - thanks to their answers - we were reassured that they were actually playing the game we intend to study.

Finally, to further support our choice of the data exclusion, we report in Figure S8 the distribution of choices in the GG in total sample, and clean sample (after removing random guesses.). As it is possible to see from the pictures below, the distributions are very similar, as observations of random choices distributed across possible values (with a relative concentration toward smaller values). Considering the motives of random guessers discussed above, this bias for lower values is likely to be due to the fact that everyday events suggest more smaller numbers than high. Further, the picture shows that we did not just eliminate non-strategic answers, but answers in all the spectrum of possible answers to the guess.

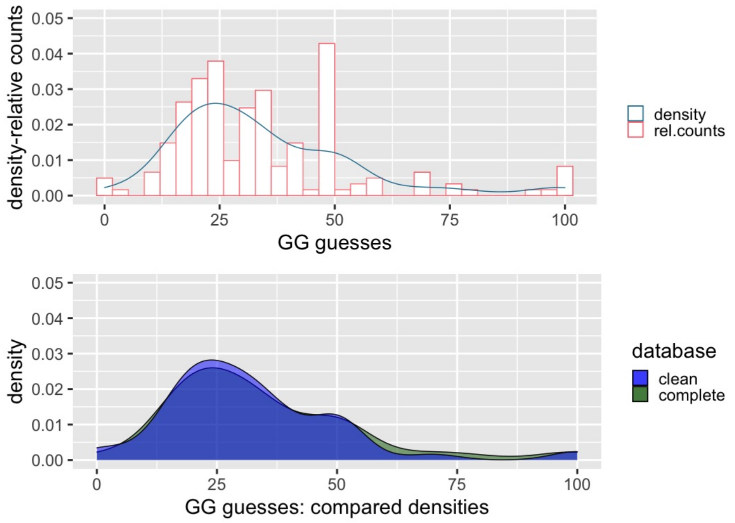

Figure S8: Top: histogram of choices in the guessing game, counts and density. Bottom: Density distribution of choices comparing complete database and clean (excluding random guesses).

S4 Literature review to support the potential connection between mentalizing, empathy and pro sociality

With our experimental setup, we find that there is a role of strategizing in the relationship between reflectivity and prosociality and we speculate that this depends from a common cognitive root of empahy and strategizing. While proving this hypothesis remains outside the scope of this paper, we propose the following steps to explore the support from the literature to this hypothesis. In order to sustain the aforementioned hypothesis, we need to discuss the literature in support of the following statements and relationships:

Strategic reasoning is the ability to predict others' mental states in order to make own decisions. Strategic reasoning is defined as the ability to think about others thoughts and mental states to predict their intentions and actions (Coricelli and Nagel, 2009). The guessing game is commonly used in the literature to investigate whether and how a player's mental process incorporates the behavior of the other players in conscious reasoning (Nagel, 1995). It is important to qualify the term strategic reasoning in relationship with mentalizing or Theory of Mind (Leslie, 1987), because the concept of strategizing is more specific to the experimental economics literature while mentalizing and Theory of Mind (ToM) are

terms used in the psychological literature. However, the two concepts are the same when considering strategic games, and in particular the guessing game, as suggested by Coricelli and Nagel (2009) : "*playing against other subjects in the Beauty Contest game activated the mentalizing network*".

Empathy and strategic thinking are related. Hooker et al. (2008) maintains that there is a natural connection between mentalizing and empathy. This interpretation is in line with what we hypothesize being the link between empathy and mentalizing in our design: while analyzing the potential outcomes of the DG game, the individual endowed with higher mentalizing abilities, would have a better ability to simulate the experience of the other which is given an unfair allocation of money, without any merit or guilt that can be ascribed to the person that receives the unfair allocation. In our design, the individual which is deciding the allocation of resources could identify himself with the subject that potentially receives an unfair share of the pie, and by projecting upon himself the potential outcome accruing to others, could elicit a (pro social) empathic response. According to Hooker et al. (2008), this ability can be inferred and does not need to be generated out of an observable experience. In their study, the authors isolate neural mechanisms involved in predicting a future emotional response of another person and investigate how activity in these neural mechanisms is related to empathy. They suggest that when people predict an emotional response in someone else, they generate an internal affective representation of the predicted emotional response. This natural ability could indicate that "*dispositionally empathic people are more practiced at generating emotion representations in the course of their relationships, thus were more likely to use emotion representations in our task*" (Hooker et al., 2008).

From these results we deduce that there is a potential neural connection between a prediction about a future emotional response of another person and empathy. We further deduce that this ability is associated with dispositionally empathic people, i.e. this ability is a natural disposition of individuals. This is what our CRT, played at the end of the experiment, aims at capturing: we use the intuitive versus reflective codification of the CRT to capture the general disposition of the person in the experiment. This is a starkly different approach with respect to that of most of the literature which consider tools to elicit an intuitive versus a reflective response (as oppose to its predisposition) in the same person (See for example Kvarven et al. (2020) for a meta-analysis).

A more recent contribution we found in support of the idea of common processes charac-

terizing ToM and Empathy is Schurz et al. (2020) which reviews papers up to 2019, including 103 studies concerning ToM literature and 85 studies related to the Empathy literature, for a total of 188 studies. Authors propose to organize the concepts of ToM and Empathy focusing on the underlying processes that characterize tasks related to both, and connect them, using common neurocognitive components engaged by different empathy and ToM tasks. To this purpose they proceed with a meta-analysis that – through a clustering tree – aims at capturing relationships among sub-components across general tasks related to Tom and Empathy. Their results suggest the presence of common neurocognitive components across mentalizing and empathy tasks, which means that empathy and TOM share specific processes and therefore brain activities. In building their 3 clusters, authors do not consider the specific task we use in our design, but we surmise that it can be considered as part of the intermediated group, which combines cognitive and affective processes[3].

Link between empathy and pro social behavior The final step that we need to explore is the one linking empathy to pro social behavior. Recent contributions, like Lockwood et al. (2013) have found a number of reasons to connect empathy with pro social behavior and we refer to these studies for a complete review (Lockwood et al., 2016; Lockwood, 2016; Lockwood et al., 2014). However, it is important to notice that Lockwood et al. (2016) identify a precise mechanistic link explaining the connection between empathy and prosocial learning i,e. through the higher ability of more empathic people to learn to benefit others. The authors tested whether individuals higher in empathy learned at a similar rate to obtain rewards for others compared with themselves and whether variability in empathy modulated neural responses to prosocial prediction errors. They find that more empathic people *learn more quickly when benefitting others*, identifying the precise link between the two processes.

This concludes our analysis of the literature performed with the aim of identifying support for empathy as the common root explaining our result that links strategic reasoning, reflectivity and pro sociality. While we find some support from the literature, this is by no means conclusive and a set of more specific experimental designs are needed to confirm or reject this hypothesis.

[3]In the cognitive cluster, the authors indicate strategic games that involve the process of decoupling (Frith and Frith, 2003) used to track down potential deception in order to solve the game. As deception is impossible in our design we can exclude this cluster. Our tasks cannot be included in the Affective Cluster either, because our tasks are in no way involved in the specific identification of empathy.

S5 Additional Statistical Analysis

This section presents additional statistical analysis concerning the relationship between pro sociality, strategic reasoning and cognitive reflection. All results reported in this section refer to the dataset where random guesses have been dropped from the dataset.

Table S11 confirms the robustness of results of Table 6 of the main text, by reproducing the OLS test with standard errors clustered at the session level.

Table S12 confirms the robustness of results of Table 7 of the main text, by reproducing the computation of theoretical and estimated coefficients for the CRT.01 specification of the relectivity (Models 1 and 3) .

In Table S13 and Table S14 we run the same regressions on the datasets that respectively include and exclude the observations related to subjects that admitted to have hazarded a guess in the Guessing Game. All Models which include an interaction term between strategic reasoning and the crt outcome (Models 4-6-7-8-9-10), present coherent results: The term *strat* is positive and significant, as well as the dummies related to the outcome of the CRT, *crt.01* and *crt.dummy* which are positive and significant. Furthermore, the interaction terms *strat:crt.01* and *strat:crt.dummy* are always negative and significant independently from the inclusion of the control variables *student, age, gender and training question.* Age is negatively related to being pro social - the older the selfish - and consistently being a student is positively related to pro sociality (as students are younger in general and in our sample). The training question is not significant showing that the results is not related to the understanding of the rules of the game. The significance of these results is confirmed in Table S15 where standard errors clustered at Session level are reported.

As a robustness check we run a ordered logistic regression and report the results in Table S16: the sign and the significance of all terms is consistent with what found in Table S14. In Table S17, S18, S19, S20, S21, S22 the odds ratios and confidence intervals for the ordered logit models are reported. Finally, in Table S23 and Table S24 we also tested our OLS estimation with a codification of the Distribution Game in two categories, defining as selfish subjects opting for choices A and B in the DG and pro social those making the choice C. Also in this case the results are consistent with our previous results, both with standard OLS and with clustering at session level. In Table S25 we run the same estimates with a logit model with clustering at session level in Table S26.

Finally, considering the large age span of the participants our experiment (from18-65-

year-old) and the relationship between age and cognitive ability which has been detected in a large number of experimental contributions, the age effect on the strategizing and reflectivity needs to be assessed. In particular it is necessary to test whether the moderate effect of strategizing on reflectivity and prosociality depends from age. For this reason, in Table S27 we regress age on each of the key dimensions of our analysis (guess, crt, pro sociality). We find that only the relationship between age and the choice in the dictator is significant and negative: older people are more likely to choose the selfish option. Interestingly (though not statistically relevant) the relationship between age and the choice in guessing game is positive, while the relationship with the CRT is negative. This means that older people tend to be less strategic and less able to engage in cognitive reflection, but not in a statistically significant way. Overall this means that the effect of age on pro sociality, observed in Table 6 of the main text is a direct effect, not significantly mediated by strategic or cognitive abilities.

Table S11: Pro sociality, strategic reasoning and reflectivity. OLS with standard errors clustered at session Level

Dep. Var. : Pro sociality	Model 1	Model 2	Model 3	Model 4
(Intercept)	1.10*	1.43***	1.94**	2.34***
	(0.64)	(0.37)	(0.93)	(0.60)
STRAT	1.67*	1.29**	1.64**	1.26**
	(0.88)	(0.59)	(0.80)	(0.52)
CRT.01	0.76*		0.72*	
	(0.42)		(0.40)	
STRAT:CRT01	−0.97*		−1.04**	
	(0.53)		(0.48)	
CRT.DUMMY		0.48**		0.47**
		(0.19)		(0.19)
STRAT:CRTDUMMY		−0.64**		−0.69***
		(0.29)		(0.25)
gender			−0.19	−0.22
			(0.22)	(0.21)
age			−0.02***	−0.02***
			(0.01)	(0.01)
training question			0.19	0.13
			(0.20)	(0.20)

Notes: The dependent variable is the outcome in the Distribution game that measures pro sociality. The term STRAT is a dummy variable taking value one for subjects with a guess lower than 50 in the GG. The term CRT.01 is a dummy taking value 1 for subjects responding correctly to at least one question in the CRT and zero otherwise. CRT.DUMMY is a dummy variable taking value 1 for subjects with no correct answers to the CRT, value 2 for those responding correctly to 2 or 3 questions, and 3 otherwise. The term *gender* is equal to 1 for male and *training question* takes value 1 for subjects responding correctly to the training question of the GG. Significance Levels: $^{***}p < 0.01$, $^{**}p < 0.05$, $^{*}p < 0.1$

Table S12: Theoretical and estimated coefficients: Models 1 and 3

Theoretical coefficients

Equation:
$\text{PRO SOC} = \alpha + b_1\,\text{STRAT} + b_2\,\text{CRT.01} + b_3\,\text{STRAT} : \text{CRT.01}$

	naïve (STRAT=0)	strategic (STRAT=1)
intuitive (CRT.01=0)	α	$\alpha + b_1$
reflective (CRT.01=1)	$\alpha + b_2$	$\alpha + b_1 + b_2 + b_3$

Calculated coefficients

	Model 1		Model 3	
	naïve	strategic	naïve	strategic
intuitive	1.10	2.77	1.94	3.58
reflective	1.86	2.56	2.66	3.26

Notes: Theoretical coefficients indicate how to calculate the coefficients related to each type of subject according to the two classifications (Strategic-reflective), using the estimated coefficients from Equation 1 (main text) and reported in Table 5 (main text). The control questions are excluded from the calculation of the coefficients because they are invariant to subjects' types by definition.

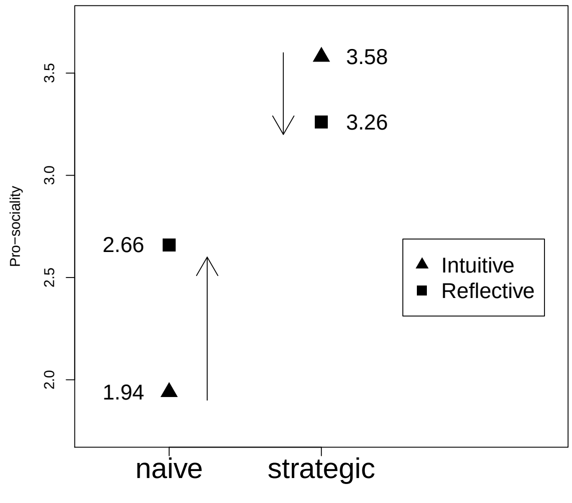

Figure S9: Estimated Coefficients Model 3 (of Table S12)

Table S13: Pro sociality. OLS. Dataset including random guesses in GG

	Model 1	Model 2	Model 3	Model 4	Model 5	Model 6	Model 7	Model 8	Model 9	Model 10
(Intercept)	2.13***	2.40***	2.18***	1.83***	2.24***	1.76***	1.59***	1.26**	1.55***	1.25***
	(0.39)	(0.41)	(0.29)	(0.48)	(0.21)	(0.35)	(0.57)	(0.55)	(0.48)	(0.46)
guess	−0.00	−0.01*								
	(0.00)	(0.00)								
crt	0.01	−0.09								
	(0.04)	(0.06)								
age	−0.00	−0.00					−0.00		−0.00	
	(0.00)	(0.00)					(0.00)		(0.00)	
gender	0.09	0.08					0.07	0.12	0.06	0.11
	(0.14)	(0.14)					(0.13)	(0.13)	(0.14)	(0.13)
training question	0.30	0.28					0.32	0.33*	0.32	0.33*
	(0.20)	(0.20)					(0.21)	(0.19)	(0.20)	(0.19)
guess:crt		0.00*								
		(0.00)								
strat			0.15	0.66	0.15	0.81*	0.75	0.84	0.80*	0.82*
			(0.14)	(0.59)	(0.15)	(0.42)	(0.60)	(0.59)	(0.43)	(0.42)
crt.01			0.04	0.25			0.26	0.33		
			(0.15)	(0.27)			(0.27)	(0.28)		
strat:crt.01				−0.29			−0.38	−0.45		
				(0.33)			(0.34)	(0.33)		
crt.dummy					0.00	0.25			0.27	0.30*
					(0.09)	(0.17)			(0.17)	(0.18)
strat:crt.dummy						−0.33*			−0.36*	−0.38*
						(0.20)			(0.20)	(0.20)
student								0.09		0.08
								(0.13)		(0.13)
AIC	416.50	414.74	436.97	438.16	437.05	436.22	417.69	425.60	415.78	423.78
BIC	438.33	439.68	449.65	454.01	449.74	452.07	442.63	450.73	440.72	448.91
Log Likelihood	-201.25	-199.37	-214.49	-214.08	-214.53	-213.11	-200.84	-204.80	-199.89	-203.89
Deviance	108.89	106.46	117.91	117.37	117.97	116.08	108.36	109.85	107.13	108.68
Num. obs.	167	167	176	176	176	176	167	171	167	171

Notes: Regressions in this table are run with the complete dataset which includes random guesses in GG. Guess: value of the guess in the GG. crt: number of correct answers to the CRT; gender=1 for male; strat: dummy equal to one for guesses in the GG smaller than 50, and zero otherwise; crt.01: dummy equal to one for subjects responding correctly to at least one question in the CRT and zero otherwise; crt.dummy: dummy taking value 1 for no correct answers, 2 for two and three correct answers, 3 otherwise. Significance Levels: $^{***}p < 0.01$, $^{**}p < 0.05$, $^{*}p < 0.1$

Table S14: Dependent variable: Pro sociality. OLS

	Model 1	Model 2	Model 3	Model 4	Model 5	Model 6	Model 7	Model 8	Model 9	Model 10
(Intercept)	3.20***	3.57***	2.11***	1.10*	2.33***	1.43***	1.94**	0.78	2.34***	1.25**
	(0.54)	(0.57)	(0.43)	(0.65)	(0.30)	(0.48)	(0.75)	(0.72)	(0.61)	(0.60)
guess	−0.00	−0.01								
	(0.00)	(0.01)								
crt	−0.03	−0.15*								
	(0.05)	(0.08)								
age	−0.02**	−0.02***					−0.02***		−0.02***	
	(0.01)	(0.01)					(0.01)		(0.01)	
gender	−0.23	−0.21					−0.19	−0.17	−0.22	−0.17
	(0.19)	(0.19)					(0.17)	(0.17)	(0.17)	(0.18)
training question	0.20	0.03					0.19	0.27	0.13	0.22
	(0.30)	(0.32)					(0.30)	(0.29)	(0.30)	(0.29)
guess:crt		0.00*								
		(0.00)								
strat			−0.01	1.67*	0.01	1.29**	1.64*	1.85**	1.26**	1.31**
			(0.21)	(0.85)	(0.21)	(0.59)	(0.85)	(0.84)	(0.59)	(0.59)
crt.01			0.14	0.76**			0.72*	0.89**		
			(0.23)	(0.38)			(0.37)	(0.38)		
strat:crt.01				−0.97**			−1.04**	−1.14**		
				(0.48)			(0.47)	(0.47)		
crt.dummy					0.01	0.48**			0.47**	0.56**
					(0.12)	(0.24)			(0.23)	(0.25)
strat:crt.dummy						−0.64**			−0.69**	−0.71**
						(0.27)			(0.27)	(0.28)
student								0.46***		0.42**
								(0.17)		(0.17)
AIC	216.30	215.04	233.17	230.96	233.55	230.04	213.08	221.69	211.35	221.57
BIC	233.56	234.76	243.30	243.62	243.68	242.71	232.81	241.68	231.07	241.57
Log Likelihood	-101.15	-99.52	-112.59	-110.48	-112.77	-110.02	-98.54	-102.84	-97.67	-102.79
Deviance	52.11	50.19	61.31	58.59	61.56	58.02	49.08	51.80	48.11	51.73
Num. obs.	87	87	93	93	93	93	87	90	87	90
num-df	5.00	6.00	2.00	3.00	2.00	3.00	6.00	6.00	6.00	6.00
den-df	81.00	80.00	90.00	89.00	90.00	89.00	80.00	83.00	80.00	83.00
effect size	0.11	0.15	0.00	0.05	0.00	0.06	0.18	0.17	0.20	0.17
significance	0.05	0.05	0.05	0.05	0.05	0.05	0.05	0.05	0.05	0.05
power	0.63	0.76	0.08	0.40	0.05	0.48	0.83	0.82	0.88	0.82
R-squared	0.10	0.13	0.00	0.05	0.00	0.06	0.15	0.15	0.17	0.15

Notes: Regressions in this table are run excluding subjects expressing a random guesses in GG (according to variable CC-GG). crt: number of correct answers to the CRT; gender=1 for male; training question: dummy variable equal to one for subjects responding correctly to the control question for the GG; strat: dummy equal to one for guesses in the GG smaller than 50, and zero otherwise; crt.01: dummy equal to one for subjects responding correctly to at least one question in the CRT and zero otherwise; crt.dummy: dummy taking value 1 for no correct answers, 2 for two and three correct answers, 3 otherwise. Significance Levels: $^{***}p < 0.01$, $^{**}p < 0.05$, $^{*}p < 0.1$. num-df and den-df indicate degrees of freedom for the numerator and denominator, effect size and significance for multiple regression power calculation.

Table S15: Dependent variable: Pro sociality. Clustered standard errors

	Model 1	Model 2	Model 3	Model 4	Model 5	Model 6	Model 7	Model 8	Model 9
(Intercept)	3.20***	3.57***	2.11***	1.10*	1.43***	1.94**	0.78	2.34***	1.25***
	(0.53)	(0.50)	(0.39)	(0.64)	(0.37)	(0.93)	(0.76)	(0.60)	(0.47)
guess	−0.00	−0.01**							
	(0.01)	(0.00)							
crt	−0.03	−0.15***							
	(0.04)	(0.05)							
age	−0.02***	−0.02***				−0.02***		−0.02***	
	(0.00)	(0.01)				(0.01)		(0.01)	
gender	−0.23	−0.21				−0.19	−0.17	−0.22	−0.17
	(0.22)	(0.20)				(0.22)	(0.19)	(0.21)	(0.18)
training question	0.20	0.03				0.19	0.27*	0.13	0.22
	(0.18)	(0.21)				(0.20)	(0.16)	(0.20)	(0.17)
guess:crt		0.00***							
		(0.00)							
strat			−0.01	1.67*	1.29**	1.64**	1.85**	1.26**	1.31***
			(0.18)	(0.88)	(0.59)	(0.80)	(0.75)	(0.52)	(0.46)
crt.01			0.14	0.76*		0.72*	0.89**		
			(0.24)	(0.42)		(0.40)	(0.38)		
strat:crt.01				−0.97*		−1.04**	−1.14**		
				(0.53)		(0.48)	(0.48)		
crt.dummy					0.48**			0.47**	0.56***
					(0.19)			(0.19)	(0.15)
strat:crt.dummy					−0.64**			−0.69***	−0.71***
					(0.29)			(0.25)	(0.23)
student							0.46***		0.42**
							(0.16)		(0.17)

Notes: OLS with standard errors clustered at Session Level. Dataset excluding random guessess in GG. Clustered Standard Errors. Guess: value of the guess in the GG. crt: number of correct answers to the CRT; gender=1 for male; strat: dummy equal to one for guesses in the GG smaller than 50, and zero otherwise; crt.01: dummy equal to one for subjects responding correctly to at least one question in the CRT and zero otherwise; crt.dummy: dummy taking value 1 for no correct answers, 2 for two and three correct answers, 3 otherwise. Significance Levels: $^{***}p < 0.01$, $^{**}p < 0.05$, $^{*}p < 0.1$

Table S16: Dependent variable: Pro sociality. Ordered logit

	Model 1	Model 2	Model 3	Model 4	Model 5	Model 6
strat	3.86*	3.10**	4.59**	5.05**	3.62**	4.46***
	(2.00)	(1.41)	(2.25)	(2.13)	(1.58)	(1.71)
crt.01	1.88**		2.11**	2.59**		
	(0.91)		(0.99)	(1.05)		
strat:crt.01	−2.36**		−3.02**	−3.27**		
	(1.17)		(1.30)	(1.29)		
crt.dummy		1.24*			1.52**	2.32**
		(0.64)			(0.68)	(0.97)
strat:crt.dummy		−1.62**			−2.09***	−2.71***
		(0.73)			(0.79)	(1.04)
gender			−0.30	−0.25	−0.39	−0.30
			(0.45)	(0.44)	(0.46)	(0.45)
age			−0.04***		−0.05***	
			(0.02)		(0.02)	
training question			0.28	0.52	0.11	0.42
			(0.78)	(0.71)	(0.77)	(0.69)
student				1.17**		1.15**
				(0.46)		(0.46)
AIC	185.50	184.80	172.09	176.89	170.33	175.11
BIC	198.16	197.46	191.82	196.89	190.05	195.11
Log Likelihood	-87.75	-87.40	-78.05	-80.45	-77.16	-79.56
Deviance	175.50	174.80	156.09	160.89	154.33	159.11
Num. obs.	93	93	87	90	87	90

Notes: Ordered logit. Dependent variable: distribution game. crt: number of correct answers to the CRT; gender=1 for male; strat: dummy equal to one for guesses in the GG smaller than 50, and zero otherwise; crt.01: dummy equal to one for subjects responding correctly to at least one question in the CRT and zero otherwise; crt.dummy: dummy taking value 1 for no correct answers, 2 for two and three correct answers, 3 otherwise. Significance Levels: $^{***}p < 0.01$, $^{**}p < 0.05$, $^{*}p < 0.1$

Table S17: Ordered Logit. Odd ratios and Confidence Intervals. Model 1

	Value	Std. Error	t value	p value	OR	2.5 %	97.5 %
strat	3.86	2.00	1.93	0.05	47.48	1.06	2966.88
crt.01	1.88	0.91	2.06	0.04	6.55	1.16	44.50
strat:crt.01	-2.36	1.17	-2.01	0.04	0.09	0.01	0.87
1|2	1.61	1.41	1.14	0.25			
2|3	2.63	1.43	1.84	0.07			

Notes: Ordered logit. Dependent variable: distribution game. strat: dummy equal to one for guesses in the GG smaller than 50, and zero otherwise; crt.01: dummy equal to one for subjects responding correctly to at least one question in the CRT and zero otherwise.

Table S18: Ordered Logit. Odd ratios and Confidence Intervals. Model 2

	Value	Std. Error	t value	p value	OR	2.5 %	97.5 %
strat	3.10	1.41	2.20	0.03	22.30	1.49	400.97
crt.dummy	1.24	0.64	1.94	0.05	3.44	1.09	14.39
strat:crt.dummy	-1.62	0.73	-2.22	0.03	0.20	0.04	0.76
1|2	0.87	1.12	0.78	0.43			
2|3	1.89	1.13	1.67	0.09			

Notes: Ordered logit. Dependent variable: distribution game. crt.dummy: dummy taking value 1 for no correct answers, 2 for two and three correct answers, 3 otherwise.

Table S19: Ordered Logit. Odd ratios and Confidence Intervals. Model 3

	Value	Std. Error	t value	p value	OR	2.5 %	97.5 %
strat	4.59	2.25	2.04	0.04	98.86	1.44	12200.16
crt.01	2.11	0.99	2.13	0.03	8.21	1.28	65.83
gender	-0.30	0.45	-0.67	0.50	0.74	0.30	1.77
age	-0.04	0.02	-2.59	0.01	0.96	0.93	0.99
training question	0.28	0.78	0.36	0.72	1.32	0.26	6.03
strat:crt.01	-3.02	1.30	-2.32	0.02	0.05	0.00	0.56
1|2	-0.01	1.70	-0.00	1.00			
2|3	1.10	1.70	0.65	0.52			

Notes: Ordered logit. Dependent variable: distribution game. crt: number of correct answers to the CRT; gender=1 for male; strat: dummy equal to one for guesses in the GG smaller than 50, and zero otherwise; crt.01: dummy equal to one for subjects responding correctly to at least one question in the CRT and zero otherwise.

Table S20: Ordered Logit. Odd ratios and Confidence Intervals. Model 4

	Value	Std. Error	t value	p value	OR	2.5 %	97.5 %
strat	3.62	1.58	2.30	0.02	37.38	1.85	957.29
crt.dummy	1.52	0.68	2.23	0.03	4.55	1.32	20.19
gender	-0.39	0.46	-0.85	0.40	0.68	0.27	1.65
age	-0.05	0.02	-2.76	0.01	0.95	0.92	0.99
training question	0.11	0.77	0.14	0.89	1.12	0.23	5.00
strat:crt.dummy	-2.09	0.79	-2.63	0.01	0.12	0.02	0.54
1|2	-1.02	1.46	-0.70	0.48			
2|3	0.11	1.45	0.07	0.94			

Notes: Ordered logit. Dependent variable: distribution game. crt: number of correct answers to the CRT; gender=1 for male; strat: dummy equal to one for guesses in the GG smaller than 50, and zero otherwise; crt.dummy: dummy taking value 1 for no correct answers, 2 for two and three correct answers, 3 otherwise.

Table S21: Ordered Logit. Odd ratios and Confidence Intervals. Model 5

	Value	Std. Error	t value	p value	OR	2.5 %	97.5 %
strat	5.05	2.13	2.37	0.02	155.42	2.76	13141.18
crt.01	2.59	1.05	2.46	0.01	13.37	1.91	132.47
gender	-0.25	0.44	-0.57	0.57	0.78	0.32	1.83
student	1.17	0.46	2.56	0.01	3.23	1.35	8.17
training question	0.52	0.71	0.74	0.46	1.69	0.40	6.83
strat:crt.01	-3.27	1.29	-2.53	0.01	0.04	0.00	0.42
1|2	3.03	1.69	1.79	0.07			
2|3	4.09	1.72	2.38	0.02			

Notes: Ordered logit. Dependent variable: distribution game. crt: number of correct answers to the CRT; gender=1 for male; strat: dummy equal to one for guesses in the GG smaller than 50, and zero otherwise; crt.01: dummy equal to one for subjects responding correctly to at least one question in the CRT and zero otherwise.

Table S22: Ordered Logit. Odd ratios and Confidence Intervals. Model 6

	Value	Std. Error	t value	p value	OR	2.5 %	97.5 %
strat	4.46	1.71	2.61	0.01	86.90	3.57	3121.55
crt.dummy	2.32	0.97	2.39	0.02	10.22	1.95	94.52
gender	-0.30	0.45	-0.66	0.51	0.74	0.30	1.78
student	1.15	0.46	2.49	0.01	3.14	1.30	7.99
training question	0.42	0.69	0.61	0.54	1.52	0.38	5.89
strat:crt.dummy	-2.71	1.04	-2.61	0.01	0.07	0.01	0.41
1|2	2.64	1.61	1.64	0.10			
2|3	3.72	1.63	2.28	0.02			

Notes: Ordered logit. Dependent variable: distribution game. crt: number of correct answers to the CRT; gender=1 for male; strat: dummy equal to one for guesses in the GG smaller than 50, and zero otherwise; crt.dummy: dummy taking value 1 for no correct answers, 2 for two and three correct answers, 3 otherwise.

Table S23: Pro sociality. Ols with dependent variable DG codified in two categories

	Model 1	Model 2	Model 3	Model 4	Model 5	Model 6
(Intercept)	-0.48	-0.11	-0.29	-0.96^{**}	0.09	-0.59^{*}
	(0.39)	(0.29)	(0.45)	(0.42)	(0.37)	(0.35)
strat	1.24^{**}	0.90^{**}	1.30^{**}	1.35^{***}	0.87^{**}	0.93^{***}
	(0.51)	(0.35)	(0.51)	(0.49)	(0.36)	(0.35)
crt.01	0.63^{***}		0.59^{***}	0.72^{***}		
	(0.23)		(0.22)	(0.22)		
strat:crt.01	-0.71^{**}		-0.79^{***}	-0.84^{***}		
	(0.28)		(0.28)	(0.28)		
crt.dummy		0.35^{**}			0.35^{**}	0.44^{***}
		(0.14)			(0.14)	(0.15)
strat:crt.dummy		-0.44^{***}			-0.46^{***}	-0.51^{***}
		(0.16)			(0.16)	(0.17)
gender			0.04	0.04	0.03	0.06
			(0.10)	(0.10)	(0.11)	(0.10)
age			-0.01^{**}		-0.01^{**}	
			(0.00)		(0.00)	
training question			0.15	0.25	0.11	0.21
			(0.18)	(0.17)	(0.18)	(0.17)
student				0.24^{**}		0.21^{**}
				(0.10)		(0.10)
AIC	134.53	135.24	124.67	126.18	125.12	126.98
BIC	147.19	147.90	144.39	146.18	144.84	146.98
Log Likelihood	-62.26	-62.62	-54.33	-55.09	-54.56	-55.49
Deviance	20.77	20.93	17.76	17.92	17.85	18.08
Num. obs.	93	93	87	90	87	90

Notes: Dependent variable: prosociality from DG outcome codified in two levels: A and B (Selfish) versus C (Pro social) crt: number of correct answers to the CRT; gender=1 for male; strat: dummy equal to one for guesses in the GG smaller than 50, and zero otherwise; crt.01: dummy equal to one for subjects responding correctly to at least one question in the CRT and zero otherwise; crt.dummy: dummy taking value 1 for no correct answers, 2 for two and three correct answers, 3 otherwise. Significance Levels: $^{***}p < 0.01$, $^{**}p < 0.05$, $^{*}p < 0.1$

Table S24: Pro sociality. Ols with dependent variable DG codified in two categories. OLS with standard errors clustered at session level

	Model 1	Model 2	Model 3	Model 4	Model 5	Model 6
(Intercept)	-0.48	-0.11	-0.29	-0.96^{**}	0.09	-0.59^{**}
	(0.30)	(0.17)	(0.54)	(0.40)	(0.41)	(0.28)
strat	1.24^{**}	0.90^{***}	1.30^{***}	1.35^{***}	0.87^{***}	0.93^{***}
	(0.50)	(0.30)	(0.42)	(0.42)	(0.29)	(0.23)
crt.01	0.63^{***}		0.59^{***}	0.72^{***}		
	(0.21)		(0.22)	(0.18)		
strat:crt.01	-0.71^{**}		-0.79^{***}	-0.84^{***}		
	(0.30)		(0.27)	(0.26)		
crt.dummy		0.35^{***}			0.35^{***}	0.44^{***}
		(0.11)			(0.13)	(0.07)
strat:crt.dummy		-0.44^{***}			-0.46^{***}	-0.51^{***}
		(0.16)			(0.16)	(0.11)
gender			0.04	0.04	0.03	0.06
			(0.15)	(0.13)	(0.15)	(0.13)
age			-0.01^{**}		-0.01^{**}	
			(0.00)		(0.00)	
training question			0.15^{**}	0.25^{***}	0.11	0.21^{*}
			(0.06)	(0.09)	(0.08)	(0.12)
student				0.24^{**}		0.21^{*}
				(0.11)		(0.11)

Notes: Standard errors clustered at session level. Dependent variable: prosociality from DG outcome codified in two levels: A and B (Selfish) versus C (Pro social). crt: number of correct answers to the CRT; gender=1 for male; strat: dummy equal to one for guesses in the GG smaller than 50, and zero otherwise; crt.01: dummy equal to one for subjects responding correctly to at least one question in the CRT and zero otherwise; crt.dummy: dummy taking value 1 for no correct answers, 2 for two and three correct answers, 3 otherwise. Significance Levels: $^{***}p < 0.01$, $^{**}p < 0.05$, $^{*}p < 0.1$

Table S25: Pro sociality. Logit with dependent variable DG codified in two categories

	Model 1	Model 2	Model 3	Model 4	Model 5	Model 6
(Intercept)	-4.79^{**}	-3.04^{*}	-4.58^{*}	-7.78^{***}	-2.45	-7.23^{***}
	(2.26)	(1.57)	(2.60)	(2.72)	(1.94)	(2.65)
strat	5.86^{**}	4.27^{**}	7.04^{**}	7.25^{**}	4.60^{**}	6.43^{**}
	(2.68)	(1.80)	(2.94)	(2.92)	(1.93)	(2.52)
crt.01	3.00^{**}		3.23^{**}	3.80^{***}		
	(1.26)		(1.33)	(1.38)		
strat:crt.01	-3.37^{**}		-4.27^{***}	-4.47^{***}		
	(1.47)		(1.63)	(1.64)		
crt.dummy		1.77^{**}			1.97^{**}	3.45^{**}
		(0.83)			(0.86)	(1.35)
strat:crt.dummy		-2.14^{**}			-2.49^{***}	-3.78^{***}
		(0.90)			(0.96)	(1.41)
gender			0.17	0.20	0.11	0.18
			(0.49)	(0.49)	(0.50)	(0.50)
age			-0.05^{**}		-0.05^{**}	
			(0.02)		(0.02)	
training question			0.99	1.37	0.68	1.19
			(0.98)	(0.93)	(0.90)	(0.89)
student				1.11^{**}		1.06^{**}
				(0.49)		(0.50)
AIC	126.40	126.89	116.37	118.30	116.90	116.89
BIC	136.53	137.02	133.63	135.80	134.16	134.39
Log Likelihood	-59.20	-59.44	-51.18	-52.15	-51.45	-51.45
Deviance	118.40	118.89	102.37	104.30	102.90	102.89
Num. obs.	93	93	87	90	87	90

Notes: Standard errors clustered at session level. Dependent variable: prosociality from DG outcome codified in two levels: A and B (Selfish) versus C (Pro social). crt: number of correct answers to the CRT; gender=1 for male; strat: dummy equal to one for guesses in the GG smaller than 50, and zero otherwise; crt.01: dummy equal to one for subjects responding correctly to at least one question in the CRT and zero otherwise; crt.dummy: dummy taking value 1 for no correct answers, 2 for two and three correct answers, 3 otherwise. Significance Levels: $^{***}p < 0.01$, $^{**}p < 0.05$, $^{*}p < 0.1$. Logit Model

Table S26: Pro sociality. Logit model with standard errors clustered at session level

	Model 1	Model 2	Model 3	Model 4	Model 5	Model 6
(Intercept)	-4.79^{**}	-3.04^{**}	-4.58	-7.78^{***}	-2.45	-7.23^{***}
	(2.26)	(1.46)	(2.82)	(2.62)	(2.16)	(2.42)
strat	5.86^{*}	4.27^{**}	7.04^{***}	7.25^{**}	4.60^{**}	6.43^{***}
	(3.08)	(2.01)	(2.67)	(2.85)	(1.86)	(2.28)
crt.01	3.00^{**}		3.23^{**}	3.80^{***}		
	(1.42)		(1.35)	(1.34)		
strat:crt.01	-3.37^{*}		-4.27^{***}	-4.47^{**}		
	(1.80)		(1.59)	(1.75)		
crt.dummy		1.77^{*}			1.97^{**}	3.45^{***}
		(1.05)			(0.96)	(1.19)
strat:crt.dummy		-2.14^{*}			-2.49^{**}	-3.78^{***}
		(1.24)			(1.10)	(1.37)
gender			0.17	0.20	0.11	0.18
			(0.68)	(0.61)	(0.67)	(0.59)
age			-0.05^{*}		-0.05^{**}	
			(0.02)		(0.02)	
training question			0.99^{**}	1.37^{**}	0.68	1.19^{**}
			(0.46)	(0.57)	(0.45)	(0.57)
student				1.11^{**}		1.06^{*}
				(0.54)		(0.55)

Notes: Standard errors clustered at session level. Dependent variable: prosociality from DG outcome codified in two levels: A and B (Selfish) versus C (Pro social). crt: number of correct answers to the CRT; gender=1 for male; strat: dummy equal to one for guesses in the GG smaller than 50, and zero otherwise; crt.01: dummy equal to one for subjects responding correctly to at least one question in the CRT and zero otherwise; crt.dummy: dummy taking value 1 for no correct answers, 2 for two and three correct answers, 3 otherwise. Significance Levels: $^{***}p < 0.01$, $^{**}p < 0.05$, $^{*}p < 0.1$. Logit Model

Table S27: Linear regression of age on guess, crt and dictator

	Dependent variable:		
	guess	crt	dictator
	(1)	(2)	(3)
age	0.180	-0.010	-0.017^{***}
	(0.145)	(0.015)	(0.006)
Constant	26.973^{***}	2.850^{***}	2.888^{***}
	(4.980)	(0.529)	(0.206)
Observations	89	89	89
Log Likelihood	-388.542	-189.029	-105.121
Akaike Inf. Crit.	781.083	382.058	214.241
Note:			*p<0.1; **p<0.05; ***p<0.01

S6 Structure of the Sample and Experimental Localities

Table S28: Characteristics of the Municipalities involved in the experiment

Municipality	Mirandola	Reggio Emilia	Vignola	Year	Source
Municipality Population	24035	171655	25244	2015	ISTAT
Population Density	174,77	702,68	1064,84	2011	ISTAT
% of Foreign Citizens	8,10	8,49	8,90	2015	ISTAT
Latitude	44° 53’ N	44° 42’ N	44° 28’ N		
City Elevation (m.s.l.)	18	58	125	2009	ISTAT
GDP per capita	20712	22015	20461	2014	MEF
Referendum turnout (%)	76,99	75,78	76,92	2016	Interior

Notes. ISTAT is the Italian Statistical institute, MEF is the Italian Ministry of Economics. Data about referendum turnout from the Italian Ministry of Interior affairs, are relative to the Italian constitutional referendum of December 2016.

Table S29: Structure of the Sample

	Mirandola	Reggio Emilia	Vignola	Total
**Gender**				
Male	31	13	17	61
Female	49	50	13	112
NA	0	1	2	3
**Age Class**				
18-25	24	38	11	73
26-35	22	11	3	36
36-45	9	6	2	17
46-55	15	4	1	20
56-65	7	2	7	16
65+	0	0	8	8
NA	3	3	0	6
**Employment status**				
Employed	49	25	8	82
Housewife-Retired-Unemployed-Unoccupied	8	5	12	25
Student	23	33	11	67
NA	0	1	1	2
**Marital Status**				
Single	49	53	17	119
Married	26	5	15	46
Separated/Divorced	3	3	0	6
Widow	2	10	3	
NA	0	2	0	2
**Nationality**				
Italian	76	57	30	163
Other	4	7	2	13
Rootedness				
Did Elementary School in the county	64	52	29	145
Mother Born in the county	47	32	24	103
Father Born in the county	52	24	25	101
**Easiness of using tablets**				
1-8	5	4	3	12
9-10	75	60	29	164
**Easiness of using Otree**				
1-8	6	4	6	36
9-10	74	60	26	140
**Instruction Clarity**				
Quite or Very Clear	78	60	30	168
Quite Unclear	1	4	2	7
Not Clear	0	0	0	0
Missing	1	0	0	1
**Num. participants**	80	64	32	176
**Sessions**				
	09-04-2016 h. 14.00	12-03-2016 h. 14.30	26-11-2015 h. 14.30	
	09-04-2016 h. 16.00	28-05-2016 h. 11.00	26-11-2015 h. 16.30	
	23-04-2016 h. 11.00	28-05-2016 h. 14.30		
	23-04-2016 h. 14.00	28-05-2016 h. 16.30		
	23-04-2016 h. 16.00			

S7 Translation of the Experiments

Page 1 - Tablet use

This page has the aim to help you to understand how the device you are using works. The screen of the device in front of you is sensitive to the touch of fingers. Scroll down the page. Since some pages contain more text than can be displayed in the screen, you need to scroll down the page.
If you place one finger on the screen of the device and try to move it vertically, the page will scroll accordingly.
Take a few minutes to become familiar with this mechanism if you don't know it well.
The test in the yellow box below is useless, it serves the only purpose to familiarize you with process of page scrolling on this device.
When you are ready for the next instructions scroll down, below the text in the yellow box.

YELLOW BOX WITH A LONG 'LOREM IPSUS' TEXT.

In addition to reading the instructions (shown in yellow boxes similar to the above ones), this study requires two types of activities:
In the first one, you are asked questions requiring you to enter a number. By clicking on the empty box close to the question, you will see a numeric keypad. You can enter numbers through it. To check if you understand, now enter number 42 as a response to the following question:

Which number comes after 41? *BOX FORM*

The last type of activity required to complete this study consists in the selection of ONE among several possible answers. In all the questions proposed in this format, you can choose only one option at a time, clicking with the tip of the finger on one of the answers.
You can choose any available answer and you can change your answer any time you want just clicking on the new answer.
Take a few minutes experience with the process of selecting responses and, when you're sure that you have understood, select the second answer to show you understand the operation.
This is an example of multiple choice question. Select the second answer.

1. This is not the correct answer
2. Click on this answer to confirm that you understand
3. Incorrect answer
4. Incorrect answer too
5. Wrong answer

Finally, this study consists of a series of pages in succession. You can change your answers whenever you want and, when you are sure of your choice on that page, you have to click on "next" to continue.
Once you have clicked on "next", you can not go back and change your choices.

When you're ready and feel confident in using this device, click on "next" to start the real study.

Page 2 - Welcome

Welcome!
First, thank you for participating in this study organized by Modena and Reggio Emilia University.
We inform you that at the end of the study you will be rewarded of 5 euro for your participation and of a further bonus depending of the choices you and other participants in this room will make. You will receive the reward at the end of the study, in private.
We ask you to turn off your phone. From this moment, no conversation among people in this room is allowed and communication with people outside the room is forbidden.
If you have doubts or questions to ask, at any time during the course of the study, please do not hesitate to raise you hand and one of us will come from you to clarify any doubts.
It's very important to follow the instructions carefully. During the instructions, we will use some numerical examples to illustrate more clearly the choices to be made. Those examples are not relevant for your final reward.
The survey consists of 5 parts. In each of these 5 parts, you'll be part of a group. Besides you, the group will consist of three other people. The composition of the group is anonymous. You will never be able to know who is part of your group as well as the others will

never know who is in theirs. The group composition will change in each part of the study. For each part the computer will do a random draw and establish the new group you will join.
During the study, you will be asked to take decisions. Each decision can be changed any time you want, before clicking "next" at the end of the page.
When you have clicked on "next", the last decision you've selected becomes the final one.
From this point on, your reward will be expressed in token/points. Every token/point is equal to 0,04 euro.
Click on "next" when you're ready to start.

FIRST PART - OMITTED
SECOND PART - OMITTED
THIRD PART - OMITTED

Page 26 - Fourth Part - Instructions

Now the computer will form NEW groups of 4 random participants.

Page 27 - Fourth Part - Instructions

Instructions

You have to choose one number between 0 and 100, with 0 and 100 included.
Differently from the previous parts, in this part there is a winner in each group.

Who is the winner

The winner is the participant who chooses the closest number to the mean of the numbers chosen by the components of the group, multiplied by 2/3.
The winner obtains 50 tokens, while the other participants do not receive anything. In case of a tie the 50 tokens are divided equally among all winners.

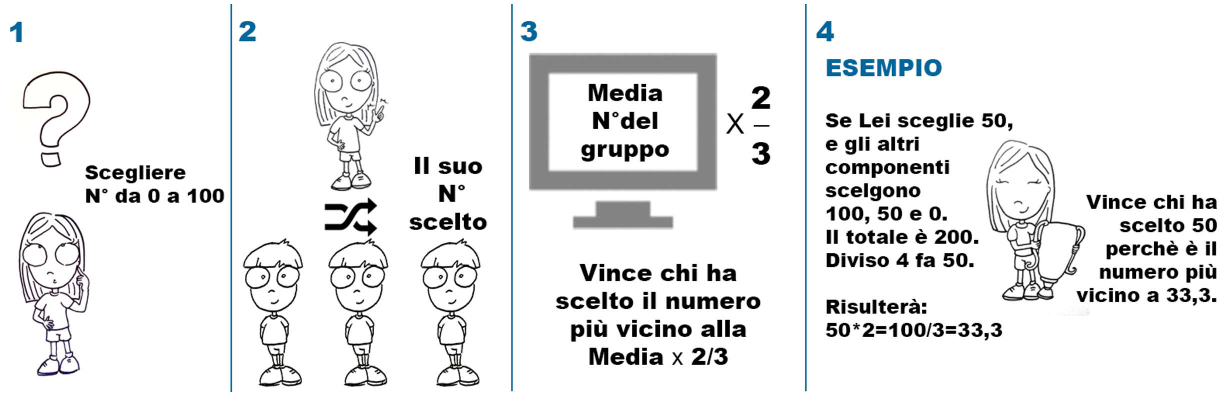

Example

Let us imagine that you choose 15, and that the other components of your group choose 25, 58 e 12. The total is: $15 + 25 + 58 + 12 = 110$. In order to obtain the mean, we need to divide this sum by 4: so 110 divided by 4, we obtain $27,5$. Then, in order to obtain the winning number, we need to multiply by $2/3$ (so we multiply the mean by 2 and divide it by 3); in our example the number to guess is then $27,5X2 = 55$ and then $55 : 3 = 18,3$ (the decimal numbers do not matter, so we say 18).

The winner is the person who guessed 15, as this is the closest number to 18.

In this case then you win because you choose 15 and you obtain 50 tokens.

Is everything clear? Are there questions?

In the next page, you will answer two questions who do not have any effect on your final payoff, but that guarantee that you correctly understood the instructions. Click “next” when you are ready.

Page 28 - Fourth Part - Training questions

Imagine you have chosen the number 20 and the other participants to your group chose the numbers 10, 20 e 70.

The mean of all numbers is 30. What is the number chosen by the winner?

The winner chose: FORM

What is your reward?

Your reward is: FORM

Page 29 - Fourth Part - Answer to the training question

Your answer '*The winner chose 0*' was wrong.

Your answer '*Your reward is: 0 tokens*' was wrong.

Imagine you have chosen the number 20 and the other participants to your group chose the numbers 10, 20 e 70. The meanof all numbers is 30. What is the number chosen by the winner? What is your reward?

Answer: The winner chose 20 and your reward is 25.

Explanation: The mean of all numbers chosen was $(20 + 10 + 20 + 70) = 120 : 4 = 30$ as indicated. The two third of 30 is equal to $30x2 = 60 : 3 = 20$.

20 is the the closest number to 20. So you and the other participant that chose 20 are the winners. Each of you receives 25 points.

Is everything clear?

Click "Next" when you are ready to proceed.

Page 30 - Fourth part - Decision

Please chose a number from 0 to 100: FORM

Page 31 - Fourth part - A comment on your choice

Now please, we ask you to write on the sheet of paper on the table a comment on your choice in this part of the study.

Did you apply a rule? If yes, could you describe shortly which one?

Click "Next" when you are ready to proceed.

Page 32 - Fifth part - Instructions

Now the computer will form NEW groups of 4 participants, chosen randomly.

Page 33 - Fifth part - Instructions

Instructions

In this part of the study you find three choices that indicate possible numbers of tokens to assign to the four members of your group.

Note that the total number of tokens assigned to your group does not change for the three choices. Only the number of the tokens assigned to each component changes.

The possible choices are the following:

[See figure S11]

What do you have to do? You have to select the choice you prefer.

How is your reward computed?

After everybody has made its choice, the computer will randomly extract one of the members of your group, that decides the rewards of all members of the group.

- If for example you are extracted, your choices will determine the rewards for all members of your group.

The person extracted, in this case you, will get the reward indicated in RED with the label YOU, while the other components of the group will get a number of tokens equal to those indicated in the image, depending on the fact that they are extracted as BLUE, GREEN or VIOLET person.

For example, If you choose A and you are extracted to decide the rewards of all components of your group, you take 30 tokens, while the others obtain 51,9 and 6 tokens, depending on the fact that they are extracted by the computer as BLUE, GREEN or VIOLET person.

- For example, if you are NOT extracted, it will be the decision of another participant to determine your reward and the one of all other members of your group.

Your reward will correspond to the reward labelled BLUE, GREEN or VIOLET person, depending on the computer's extraction.

For example, if the person extracted by the computer to decide the rewards of all members

of the group indicated Choice C, that person obtains 24 tokens, if you have been extracted by the computer as VIOLET person you get 12 tokens, the other two members of the group obtain instead 18 and 42 tokens.

The instructions will remain available on the screen for the duration of this part of the study.

Do not hesitate to raise your hand and ask questions. One of us will come to you to answer them.

Before making your choice, we ask you to answer two questions that WILL NOT HAVE ANY EFFECT on your final reward, but that guarantee that you correctly understood the instructions.

Click "Next" when you are ready to proceed.

Page 34 - Fifth part - Training Questions

(Figure S11 reproduced here)

Imagine you have chosen *Choice B*.

If you are selected by the computer to decide the rewards of everybody your reward is:

1. 15 tokens
2. 27 tokens

Imagine you have chosen *Choice A*.

If another member of your group is extracted to decide the rewards of all members of the group and that person has chosen *Choice C*. In this case your reward is:

1. 42 or 18 or 12 tokens, according to the random extraction of the computer.
2. 45 or 15 or 9 tokens, according to the random extraction of the computer.

Page 35 - Fifth part - Answer to the Training Questions

(Figure S11 reproduced here)

Your answer '*You have chosen B. If your are selected by the computer to decide the rewards of everybody your reward is: 15 tokens*' was wrong.
Your answer '*If another component of your group is extracted to decide the rewards of everybody and this person chooses C, in this case your reward is: 42 or 18 or 12 tokens, according to the random extraction of the computer*' was correct.

Answer to the first question: If you are chosen by the computer to decide the rewards of all members of your group and you choose B, then you will obtain the reward indicated in RED with the label YOU, thus 27 tokens. The other three components will obtain respectively 45, 15 and 9 tokens, depending on them being extracted as the BLUE, GREEN or VIOLET person.

Answer to the second question: On the contrary, if the computer extracts another component of your group to decide the rewards of all members of the group, and this component has chosen C, you can obtain 42, 18 or 12 tokens, depending on the fact that you are extracted as the BLUE, GREEN or VIOLET person.

Page 36 - Fifth part - Decision

(Figure S11 reproduced here)

Now you can take your decision:

1. Choice A
2. Choice B
3. Choice C

Page 37 - Fifth part - A comment on your choice

Now please, we ask you to write on the sheet of paper on the table a comment on your choice in this part of the study.
Did you apply a rule? If yes, could you describe shortly which one?

Click “Next” when you are ready to proceed.

Page 38 - Your results

Thanks for participating to this study, in the following you can find your results.

{REPORT RESULTS FIRST PART - OMITTED}

{REPORT RESULTS SECOND PART - OMITTED}

{REPORT RESULTS THIRD PART - OMITTED}

FOURTH PART

You have chosen X [Xs represent the numerical information presented to the participant]. The others have chosen X, X and X. The two third of the mean was X and the closest number was X. There are X participants that have chosen this number and you are one of them. Consequently, you receive X tokens.
For this part of the game you will be paid: X,XX €

FIFTH PART

You have chosen X.
Someone else has been selected as Person 2.
You have been selected as Person 1.
Thus, your reward for this part is XX tokens.
For this part of the game you will be paid: X,XX €

PARTICIPATION REWARD

As reward for your participation, you will further receive a fix amount of: €5.00
THE TOTAL REWARD THAT WILL BE PAID TO YOU IS: €XX,XX

BEFORE LEAVING:

Thank you for your availability. We ask you a last effort and patience while we prepare the

payments of the rewards. While you are waiting, we ask you to answer to a short questionnaire. The information provided, as well as the results of the whole study, will be managed with the upmost respect for your privacy and used solely for scientific purposes.

QUESTIONNAIRE

Questionnaire 1 - Clarity of the study

Thank you very much again for the participation to this study. As this experience draws to an end we would like to ask you a few questions:

Were the instructions of the todays' study clear?

1. Absolutely not clear
2. Not very clear
3. Quite Clear
4. Very Clear

This study has been realized with the support of tablets such as the one you keep in your hands. We would like your opinion on the easiness of using this tablet from your point of view. We ask you to indicate the answer on a scale where 1 corresponds to "Absolutely difficult to use" and 10 corresponds to "Absolutely easy to use". (Radio Button Answer from 1 to 10).

1. Absolutely difficult to use

...

10. Absolutely easy to use

We would like to have your opinion on the software interface used for this study. We ask you to indicate the answer on a scale where 1 corresponds to "Absolutely difficult to use" and 10 corresponds to "Absolutely easy to use". (Radio Button Answer from 1 to 10).

1. Absolutely difficult to use

...

10. Absolutely easy to use

Questionnaire 2 - Citizenship, Age and sex

What is your country of citizenship? Choice from drop down list

Age? Form

Sex Male or Female

Questionnaire 3 - Questionnaire

Please indicate your birthplace:

1. In this city or in the neighbouring municipalities.
2. Within the province but outside this city or the neighbouring municipalities
3. Outside the province, but within Emilia-Romagna.
4. In Trentino AA, Veneto, Lombardia, Liguria (excluding the neighbouring municipalities)
5. Toscana, Umbria, Marche, Lazio
6. Abruzzo, Molise, Puglia, Basilicata, Calabria, Campania
7. Sicilia, Sardegna
8. Abroad

Please indicate the birthplace of YOUR MOTHER:

{Same possible answers that in the question about own birthplace }

Please indicate the birthplace of YOUR FATHER:

{Same possible answers that in the question about own birthplace }

Where you were going at Elementary School?

{Same possible answers that in the question about own birthplace }

Questionnaire 4 - Earthquake 2012

When the earthquakes of the May-June 2012 struck, were you living in the area hit by the earthquake?

1. No
2. Yes

Was your main house damaged as consequence of the earthquake?

1. No, No damages
2. A - Immediately accessible
3. B - temporarily unfit for use, but accessible with measures small interventions
4. C - partially unfit for use
5. D - temporarily unfit for use, to be checked by a specialist.
6. E - unfit for use
7. F - unfit for use for external risks

[NOTE: The classification in the points A to F refer to the official civil protection classification. Each house in the area struck by the earthquake was classified by structural engineers and the result communicated to families, which are thus well informed about the classification system.]

Today, your main house is:

1. Fit for use
2. Being fixed.
3. Not yet fixed.

In the emergency period, after the earthquake:

1. I kept living in my house 2. I moved in another house within the area hit by the earthquake
3. I used the temporary structures provided by the civil protection
4. I move temporarily my residence outside the area hit by the earthquake
5. I move permanently my residence outside the area hit by the earthquake

Questionnaire 5 - Family

Marital status:

1.Unmarried

2.Married

3.Separated

4.Divorced

5.Widower

How many people compose your family, including yourself?

1.1

2.2

3.3

4.4

5.5 or more

Are you the family's main source of income?

1.Yes

2.No

Do you have (or had) brothers and/or sisters?

1.No

2.1

3.2

4.3 or more

Questionnaire 6 - House and Automobiles

Does your family own a car?

1.Yes, 1

2.Yes, 2 or more

3.No

Is the house where your family lives:

1.Rented

2.Your property

3.Other

Questionnaire 7 - Employment

Employment:

1.Inexperienced worker

2.Experienced labor force

3.Self-employed

4.Employed with fixed term contract

5.Employed with permanent contract

6.Retired

7.Student

8. Unemployed

9.Not able to work or not searching for a job

Questionnaire 8 - Taking profit

Do you think that most people would try to take advantage of you if they got a chance, or would they try to be fair? Please choose one answer in the scale from one to 10, where 1 means that "people would try to take advantage of you", and 10 means that "people would try to be fair":

1.People would try to take advantage of you

10. People would try to be fair

Questionnaire 9 - Trust in institutions

I am going to name a number of organizations. For each one, could you tell how much confidence you have in them: is it a great deal of confidence, quite a lot of confidence, not very much confidence or none at all?(one answer for each):

1.Associations or groups related to the churches

2.Sport or leisure associations or organizations

3.Artistic, music or educational associations or organizations

4.Labor Unions

5.Political Parties

6.Environmental organizations

7.Professional organizations

8.Charitable or humanitarian organizations

9.Consumers organizations

10. European Union

Questionnaire 10 - Elections, referendum and blood donations

Have you made a blood donation in the last 12 months?

1.Yes

2.No

3.I cannot donate for medical reasons

Did you vote in the last European elections in 2014?

1.Yes

2.No

3.I had no voting right

Did you vote in the last referendum of 2014?

1.Yes

2.No

3.I had no voting right

Questionnaire 10 - Studied and Worked abroad

Have you ever studied in a school or university abroad? (One of the following answers)

Have you ever worked abroad with an independent activity or had been a employee of a foreign company abroad? (One of the following answers)

1. No
2. Yes, for less than 3 months
3. Yes, for a period between 3 and 12 months

4. Yes, for a period between 12 and 36 months
5. Yes, for a period of more than 36 months

Cognitive Reflection Test

See Section S9.

Final Summary

Thanks for having participated to this study.

The study is now finished, your results for each part of the study as well as the total amount of money that you will receive are summarized below:

REWARD FOR THE FIRST PART €X,XX

REWARD FOR THE SECOND PART €X,XX

REWARD FOR THE THIRD PART €X,XX

REWARD FOR THE FOURTH PART €X,XX

REWARD FOR THE FIFTH PART €X,XX

REWARD FOR PARTICIPATION €5.00

THE TOTAL THAT WILL BE PAID TO YOU IS: €XX,XX

You are now at the end of this study,

Please remain seated until your number is called.

Your unique identifier is: XXXXXX

S8 Choice screens vignettes

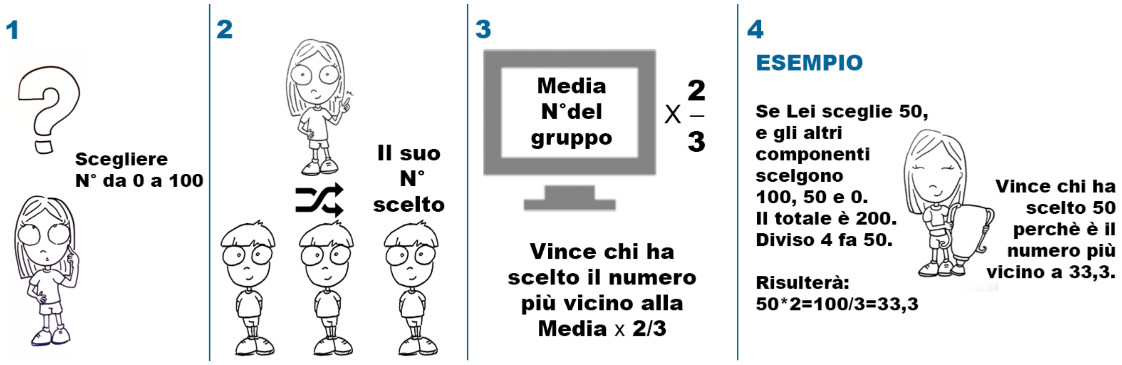

Figure S10: Guessing game choice screen

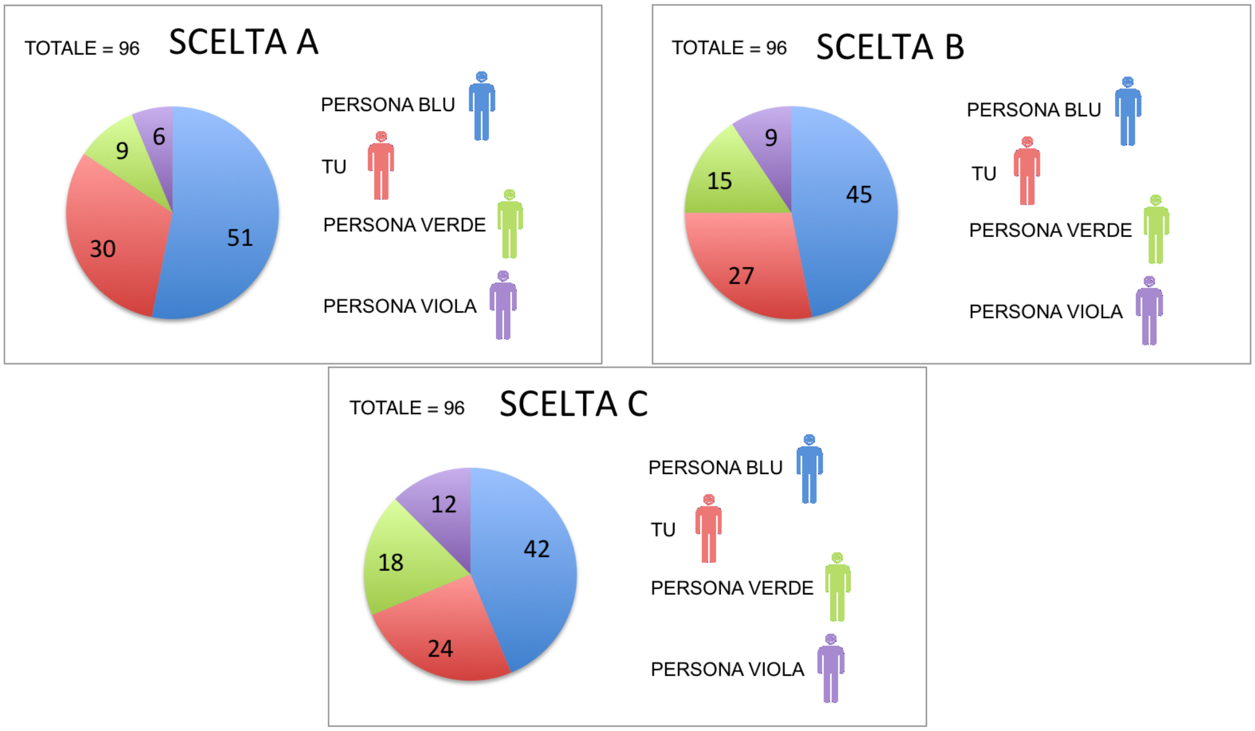

Figure S11: Dictator game choice screen

S9 Extended CRT questions from Primi et al. 2016

Please answer the following questions:

1. If you flipped a fair coin three times, what is the probability that it would land "Heads" at least once?. . . percent

2. A bat and a ball cost $1.10 in total. The bat costs $1.00 more than the ball. How much does the ball cost? …cents

3. If it takes 5 minutes for five machines to make five widgets, how long would it take for 100 machines to make 100 widgets? …minutes

4. In a lake, there is a patch of lily pads. Every day, the patch doubles in size. If it takes 48 days for the patch to cover the entire lake, how long would it take for the patch to cover half of the lake?…days

5. If three elves can wrap three toys in hour, how many elves are needed to wrap six toys in 2 hours? …elves

6. Ellen and Kim are running around a track. They run equally fast but Ellen started later. When Ellen has run 5 laps, Kim has run 15 laps. When Ellen has run 30 laps, how many has Kim run? …laps

7. Jerry received both the 15th highest and the 15th lowest mark in the class. How many students are there in the class?…students

8. In an athletics team, tall members are three times more likely to win a medal than short members. This year the team has won 60 medals so far. How many of these have been won by short athletes?…medals

S10 Parameters of the Distribution Game

Table S30: Parameters of the Distribution Game

Player	Choice A	Choice B	Choice C
Person 1	51	45	42
DICTATOR	30	27	24
Person 3	9	15	18
Person 4	6	9	12
Total Income	96	96	96
Criteria			
Population Variance	328.5	189	126
Bolton-Ockenfels (ERC)	-6.25	-3.13	0
F&S Strict	-22	-16	-12
Minimax	6	9	12
Middle Person 1-3-4	22	23	24

Notes: Population Variance is the variance of the payoffs of each choice. Bolton & Ockenfels(ERC) is calculated as $ERC = -abs\left(\frac{DictatorPayoff}{96} - \frac{1}{4}\right)$. $F\&S\,Strict = -\frac{1}{4}\sum abs(Payoff_i - DictatorPayoff)$. Minimax is the value of the minimum payoff among the four components of the group in each presented possible choice. Middle is the simple mean of the payoffs of the group in each choice excluding the dictator.

References

Coricelli, G. and R. Nagel (2009). Neural correlates of depth of strategic reasoning in medial prefrontal cortex. *Proceedings of the National Academy of Sciences 106*(23), 9163–9168.

Frith, U. and C. D. Frith (2003). Development and neurophysiology of mentalizing. *Philosophical Transactions of the Royal Society of London. Series B: Biological Sciences 358*(1431), 459–473.

Hooker, C. I., S. C. Verosky, L. T. Germine, R. T. Knight, and M. D'Esposito (2008). Mentalizing about emotion and its relationship to empathy. *Social cognitive and affective neuroscience 3*(3), 204–217.

Kvarven, A., E. Strømland, C. Wollbrant, D. Andersson, M. Johannesson, G. Tinghög, D. Västfjäll, and K. O. R. Myrseth (2020). The intuitive cooperation hypothesis revisited: a meta-analytic examination of effect size and between-study heterogeneity. *Journal of the Economic Science Association 6*(1), 26–42.

Leslie, A. M. (1987). Pretense and representation: The origins of theory of mind. *Psychological review 94*(4), 412.

Lockwood, P. L. (2016). The anatomy of empathy: Vicarious experience and disorders of social cognition. *Behavioural brain research 311*, 255–266.

Lockwood, P. L., M. A. Apps, V. Valton, E. Viding, and J. P. Roiser (2016). Neurocomputational mechanisms of prosocial learning and links to empathy. *Proceedings of the National Academy of Sciences 113*(35), 9763–9768.

Lockwood, P. L., G. Bird, M. Bridge, and E. Viding (2013). Dissecting empathy: high levels of psychopathic and autistic traits are characterized by difficulties in different social information processing domains. *Frontiers in human neuroscience 7*, 760.

Lockwood, P. L., A. Seara-Cardoso, and E. Viding (2014). Emotion regulation moderates the association between empathy and prosocial behavior. *PloS one 9*(5), e96555.

Lovie, P. (2014). Interaction plot. *Wiley StatsRef: Statistics Reference Online*.

Nagel, R. (1995). Unraveling in guessing games: An experimental study. *American Economic Review 85*(5), 1313–1326.

Schurz, M., J. Radua, M. G. Tholen, L. Maliske, D. S. Margulies, R. B. Mars, J. Sallet, and P. Kanske (2020). Toward a hierarchical model of social cognition: A neuroimaging meta-analysis and integrative review of empathy and theory of mind. *Psychological Bulletin*.
